# Flexible 3D *Kirigami* Probes for In Vitro and In Vivo Neural Applications

**DOI:** 10.1002/adma.202418524

**Published:** 2025-04-14

**Authors:** Marie Jung, Jamal Abu Shihada, Simon Decke, Lina Koschinski, Peter Severin Graff, Sebastián Maruri Pazmino, Anke Höllig, Henner Koch, Simon Musall, Andreas Offenhäusser, Viviana Rincón Montes

**Affiliations:** ^1^ Bioelectronics Institute of Biological Information Processing‐3 Forschungszentrum Jülich Jülich Germany; ^2^ Department of Physics RWTH Aachen University Aachen Germany; ^3^ Institute for Zoology RWTH Aachen University Aachen Germany; ^4^ Helmholtz Nano Facility (HNF) Forschungszentrum Jülich Jülich Germany; ^5^ Department of Neurosurgery Medical Faculty RWTH Aachen University Aachen Germany; ^6^ Department of Epileptology Neurology RWTH Aachen University Hospital Aachen Germany; ^7^ Faculty of Medicine Institute of Experimental Epileptology and Cognition Research University of Bonn Bonn Germany

**Keywords:** 3D microelectrode arrays, flexible electronics, neural implants, neurotechnology

## Abstract

3D microelectrode arrays (MEAs) are gaining popularity as brain–machine interfaces and platforms for studying electrophysiological activity. Interactions with neural tissue depend on the electrochemical, mechanical, and spatial features of the recording platform. While planar or protruding 2D MEAs are limited in their ability to capture neural activity across layers, existing 3D platforms still require advancements in manufacturing scalability, spatial resolution, and tissue integration. In this work, a customizable, scalable, and straightforward approach to fabricate flexible 3D *kirigami* MEAs containing both surface and penetrating electrodes, designed to interact with the 3D space of neural tissue, is presented. These novel probes feature up to 512 electrodes distributed across 128 shanks in a single flexible device, with shank heights reaching up to 1 mm. The 3D *kirigami* MEAs are successfully deployed in several neural applications, both in vitro and in vivo, and identified spatially dependent electrophysiological activity patterns. Flexible 3D *kirigami* MEAs are therefore a powerful tool for large‐scale electrical sampling of complex neural tissues while improving tissue integration and offering enhanced capabilities for analyzing neural disorders and disease models where high spatial resolution is required.

## Graphical abstract

A customizable and scalable approach to fabricate flexible 3D *kirigami* microelectrode arrays (MEAs) featuring up to 128 shanks, including surface and penetrating electrodes designed to interact with the 3D space of neural tissue, is presented. The 3D *kirigami* MEAs are successfully deployed in several neural applications, both in vitro and in vivo, and identified spatially dependent electrophysiological activity patterns.

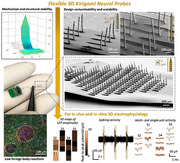

## 1 Introduction

Unveiling the intricate networks of the central nervous system and their functions in both healthy and pathological states requires investigating neural activity with high spatiotemporal resolution across multiple topological levels. This includes examining activity from individual neurons to local and regional dynamics within distinct neural structures and encompasses the 3D space of nervous tissues, extending from its surface to its complex, multilayered internal structure. Neuroelectronic interfaces comprising microelectrode arrays (MEAs) facilitate this by capturing electrical signals with sub‐milliseconds resolution and covering spatial regions from micrometers to millimeters. MEAs enable the recording of complementary neural information by capturing high‐frequency signals associated with action potentials (also known as spiking activity), and low‐frequency signals related to local field potentials (LFPs), which represent the activity of individual neurons and the collective activity of neuronal populations in localized neural areas, respectively. Consequently, MEAs are commonly used to record extracellularly and modulate the electrical activity of various neural structures in basic neuroscience and preclinical and clinical settings. Neuroscientific applications of MEAs range from understanding the neural basis of memory,^[^ ^1^ ^]^ space,^[^ ^2^ ^]^ and behavior^[^ ^3^ ^]^ to studying neural oscillations in epilepsy research^[^ ^4^ ^]^ and developing brain–machine interfaces (BMIs) to restore lost sensorimotor functions.^[^ ^5^ ^]^

MEAs containing grids of microelectrodes that are spatially arranged in two dimensions on stiff or flexible flat surfaces (2D MEAs) are widely used for surface in vitro neural recordings in neural slices,^[^ ^6^, ^7^ ^]^ as well as in vivo applications interfacing with the central and peripheral nervous system.^[^ ^8^, ^9^ ^]^ However, 2D MEAs are limited to capturing 2D spatial information. To access the deeper intraneural space, penetrating MEAs are employed,^[^ ^10^, ^11^, ^12^, ^13^, ^14^ ^]^ but they often suffer from limited 3D spatial coverage by typically capturing information from a single neural layer across a millimeters‐wide region or multiple neural layers at one spatial location.

To interact with the 3D neural space, various types of 3D MEAs have been developed to penetrate and access different neural layers and regions. Silicon‐based Utah and Michigan arrays have long been the gold standard for BMIs. Innovations such as the Utah slant array^[^ ^10^, ^15^ ^]^ and the stacking of 2D Michigan arrays^[^ ^16^, ^17^, ^18^, ^19^ ^]^ have enabled 3D spatial sampling. However, these devices often trigger strong foreign body reactions (FBRs) due to their mechanical incompatibility with neural tissue.^[^ ^20^, ^21^ ^]^ Recent advancements have led to the development of softer, more flexible (e.g., thin‐film polymers), and stretchable materials (e.g., conductive hydrogels), enabling microscale electrodes that minimize tissue damage.^[^ ^22^, ^23^, ^24^, ^25^ ^]^ These devices, however, typically involve the individual implantation of multiple threads or shanks, leading to complex surgical procedures.^[^ ^26^, ^27^, ^28^ ^]^ While flexible needle‐like probes with varying heights and multisite designs have been demonstrated,^[^ ^29^, ^30^ ^]^ increasing electrode density remains limited by the resolution and scalability of current additive manufacturing processes proposed in these works.

A potential approach to overcome these existing challenges is the development of multicontact and fully flexible 3D MEAs. Several research groups have proposed *kirigami*‐based methodologies to achieve this goal. Inspired by the Japanese art of cutting and folding paper, *kirigami* techniques are adapted to micromachining by first processing a 2D flexible MEA. The 2D MEA is then etched to create cut‐out patterns, allowing the shanks to be lifted into a 3D configuration. Various folding methods have been explored, including the use of ferromagnetic sheaths to induce folding via magnetic fields,^[^ ^31^ ^]^ as well as electrostatic^[^ ^32^ ^]^ or mechanical actuation, such as manual bending^[^ ^33^, ^34^ ^]^ for selective shank folding or stress‐induced bending for nanowire‐based transistor probes.^[^ ^35^, ^36^ ^]^ However, current limitations hinder the scalable implementation of these devices. Fabrication challenges, such as manual shank lifting, bending of high‐aspect‐ratio structures, and magnetic actuation, are time‐consuming and can lead to inconsistent folding. Additionally, the use of ferromagnetic materials, such as nickel, requires strong magnetic fields (100–400 mT) that may pose safety risks.^[^ ^31^ ^]^ These methods can also result in inconsistent folding angles or shanks folding back after implantation, compromising the precision and reliability of the 3D structure.^[^ ^33^ ^]^

To overcome the limitations of existing 3D MEAs, we developed a scalable *kirigami*‐based approach to fabricate flexible, biocompatible, and high‐resolution neural interfaces. Our method utilizes a parallelized matched die forming process, a scalable method to produce 3D MEAs with up to 128 shanks. We demonstrate the versatility of our technique by fabricating various 3D MEA designs for diverse neural applications, including in vitro and in vivo validations. Our devices enable the recording of seizure‐like activity in human brain slices and sensory responses in the living mouse brain, showcasing their potential for advancing neuroscience research.

## 2 Results and Discussion

### 2.1 Fabrication of 3D Flexible *Kirigami* Neural Probes

**Figure 1:**
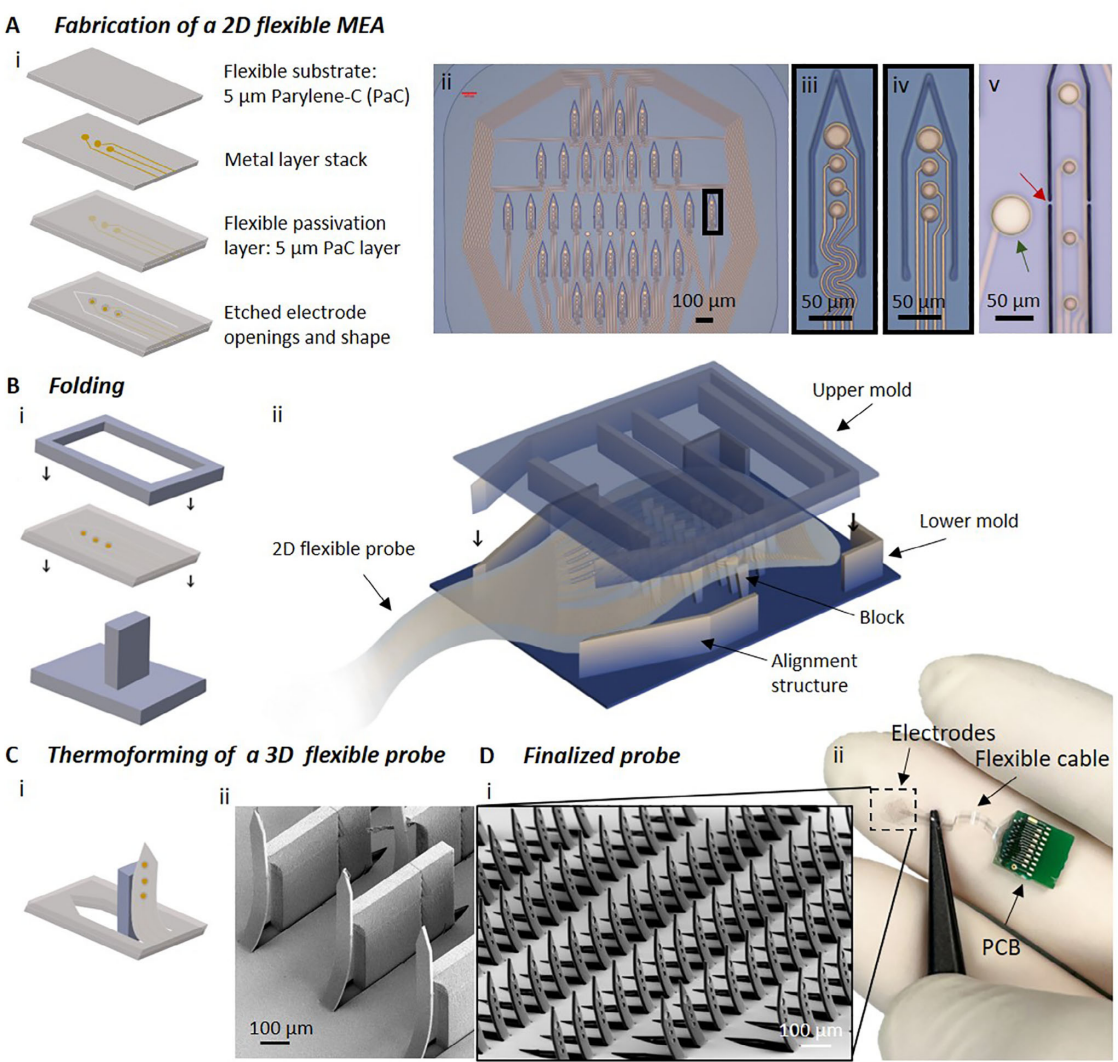
Fabrication of 3D *kirigami* MEAs. A) The fabrication of the 2D *kirigami* template comprises four steps (A_i_): 1) Chemical vapor deposition (CVD) of a 5 µm PaC layer (in gray). 2) Deposition of a Ti/Au/Ti metal layer stack (in yellow). 3) 2nd CVD process for a PaC passivation layer. 4) Reactive ion etching (RIE) to open the electrodes and etch the shape. Exemplary design aimed at retinal applications with 32 shanks (A_ii_) containing meander‐shaped (A_iii_) or straight feedlines (A_iv_). Additionally, surface electrodes (A_v_, green arrow) and breaking points for long and/or thin shanks (A_v_, red arrow) are included in the design. B) For the folding of the shanks, the 2D *kirigami* template is placed between a lower and an upper mold (one shank displayed in (B_i_) and complete probe in (B_ii_)) folding all shanks at the same time by blocks, which are arranged at each shank location and creating homogenous pressure over the complete flexible template using the upper mold. C) The flexible probe placed inside the lower mold (one shank in (C_i_) and scanning electron microscopy (SEM) picture of several shanks in (C_ii_)) is then thermoformed. D) The finalized probe (SEM picture in (D_i_)) bonded on a PCB in comparison to a human hand (ii).

We developed a process to reliably fabricate 3D flexible MEAs based on the *kirigami* principle of cutting and folding a 2D design into a 3D structure using flexible polymeric materials, such as parylene‐C (PaC). The process consists of three main steps: fabrication of a 2D *kirigami* template (**Figure** **1**A), folding of the 2D *kirigami* template into its 3D conformation (Figure 1B), and thermoforming of the 3D flexible structure (Figure 1C). To fabricate the 2D *kirigami* template, we employed a mechanically robust 2D *kirigami* design and utilized standard surface micromachining processes and thin‐film technology. We then used an additive manufacturing process to fabricate a customized mold containing upright structures that matched the location of the penetrating shanks in the 2D design. The mold enabled a matched die forming system to align and fold the 2D *kirigami* template in the third dimension. Lastly, the device was thermoformed to maintain the newly formed 3D structure. This process stands out as it allows scalability toward the parallel folding of multiple shanks (up to 512 tested so far) with a yield of 98%, avoiding the use of toxic materials or hazardous processes as proposed by other works.^[^ ^31^, ^32^ ^]^ Likewise, the process offers design customizability and flexibility, making it possible to adapt designs across a diverse range of spatial resolutions and neuronal models for both in vitro and in vivo applications.

#### 2.1.1 2D *Kirigami* Template

The process starts with the fabrication of a 2D *kirigami* template, which features a flexible sandwich structure that ultimately forms a flexible 2D MEA. This structure is composed of two PaC layers that serve as substrate and encapsulation, and a metal stack layer of Ti/Au/Ti in the middle that shapes the electrical feedlines and electrodes (Figure 1A_i_). The design of the overall probe included a contact pad region, a flexible cable, and a sensing area containing surface electrodes and penetrating shanks, each holding multiple microelectrodes (Figure S1, Supporting Information). Besides the standard electrode and contact pad openings and the outline of the implant, the 2D pattern contained the cut‐outs corresponding to the structures to be folded upright in the third dimension (Figure 1A_ii–iv_). The 2D design was then patterned via photolithography and further etched via dry etching, resembling the drawing and cutting steps of the *kirigami* process.

For a successful parallelized *kirigami* approach, the design of the 2D pattern served as a template for the 3D structure to be formed and also provided mechanical stability during the folding process. To reduce mechanical stresses at the folding sites of each shank, meander‐shaped feedlines were integrated into the design at the kink (Figure 1A_iii_) as an alternative to straight feedlines (Figure 1A_iv_), and breaking points were added at the sides of each shank to provide mechanical stability to the 2D template (Figure 1A_v_, red arrow). The latter design strategy was critical for the success of the matched die forming process with long shanks above 300 µm and thin film layers below 10 µm, as it prevents high‐aspect ratio and thin structures from failing due to self‐bending or moving out of place due to electrostatic forces. Additionally, to increase the spatial resolution of the implant, electrodes were added on the plane surface of the 2D *kirigami* template next to or between the shanks.

#### 2.1.2 Folding of the 2D *Kirigami* Template

After microfabrication, the 2D *kirigami* template, also referred to as 2D flexible MEA, was flip‐chip bonded onto a printed circuit board (PCB). The device was then ready for folding. Here, customized molds that matched the design of the 2D *kirigami* template were fabricated by two‐photon lithography. Using complementary dies, hereafter referred to as upper and lower molds, we implemented a matched die forming process (Figure 1B; Figure S2, Supporting Information). The lower mold contained protruding blocks that exactly matched the location and geometry of the shanks in the 2D *kirigami* template and sidewalls performing as alignment structures that matched the outline of the sensing area of the 2D flexible MEA (Figure 1B_i_,_ii_). For short shanks, the block height surpassed the shank length, resulting in block height‐to‐shank length ratio of 1.11 (e.g., 250 µm block height for 225 µm shank length). In contrast, for longer shanks, the blocks were shorter than the shank length, leading to ratio below 1 (e.g., 750 µm block height for 1000 µm shank length). To allow tolerance for the removal of the mold in further steps, the width of the blocks was 0.9–0.92 times the width of the shanks. Additionally, ramped side‐walled blocks were implemented to reduce the contact surface area between the blocks and the shanks, facilitating the releasing step (e.g., 120 µm at the top, 60 µm at the bottom).

This mold design allows the straightforward placement of the 2D *kirigami* template into the lower mold without requiring a complex alignment setup. Complementary to the lower mold and the 2D *kirigami* template, the upper mold contained straight protruding structures perpendicular to the planar axis of the shanks and located between rows of shanks and along the outer boundaries, thereby ensuring homogenous pressure on the 2D *kirigami* template when pressed toward the lower mold. The outer boundaries of the upper mold fit perfectly inside the lower mold (Figure 1B_ii_, tolerance to each side 35 µm), which ensures facile alignment, and together with the matching design of the lower mold, a matched die compression is enabled, allowing the concurrent folding of all shanks (Video S1, Supporting Information).

#### 2.1.3 Thermoforming of a 3D *Kirigami* MEA

Given that PaC, a thermoplastic, was used as the main substrate and encapsulation material, the flexible probe was subjected to a thermoforming step while still placed inside the lower mold after the folding (Figure 1C_i,ii_). Thermoplastics are moldable materials when subjected to temperatures above their glass transition temperature (e.g., 90 °C for PaC) and below their melting point (e.g., 290 °C for PaC).^[^ ^37^ ^]^ At high temperatures, the chain structures of the amorphous regions of the PaC undergo rearrangement, softening the material and enabling the reshaping of its molecular structure. The polymeric material then retains the new shape upon cooling and removal of the mold. The thermoforming protocol was optimized considering the trade‐off between temperature and thermoforming time. While higher temperatures allowed proper thermoforming, they caused the material to stick to the molds. On the other hand, the use of lower temperatures implied longer thermoforming periods that in most cases were not fully met, causing then the shanks to fold back immediately after the separation from the mold. In the case of PaC, it had to be considered that high temperatures (>300 °C) permanently change the mechanical and optical properties of the material.^[^ ^38^ ^]^ Hence, a thermoforming protocol was established using a slow temperature ramp of 4 °C per minute from room temperature to 160 °C, a dwelling time of 60 min at 160 °C, and a cooling step with a slow ramp to cool down until room temperature was reached. Then, the 3D flexible *kirigami* MEA and the lower mold were separated using droplets of water to facilitate the separation.

### 2.2 Variants, Limitations, and Yield of the *Kirigami*‐Based Process

**Figure 2:**
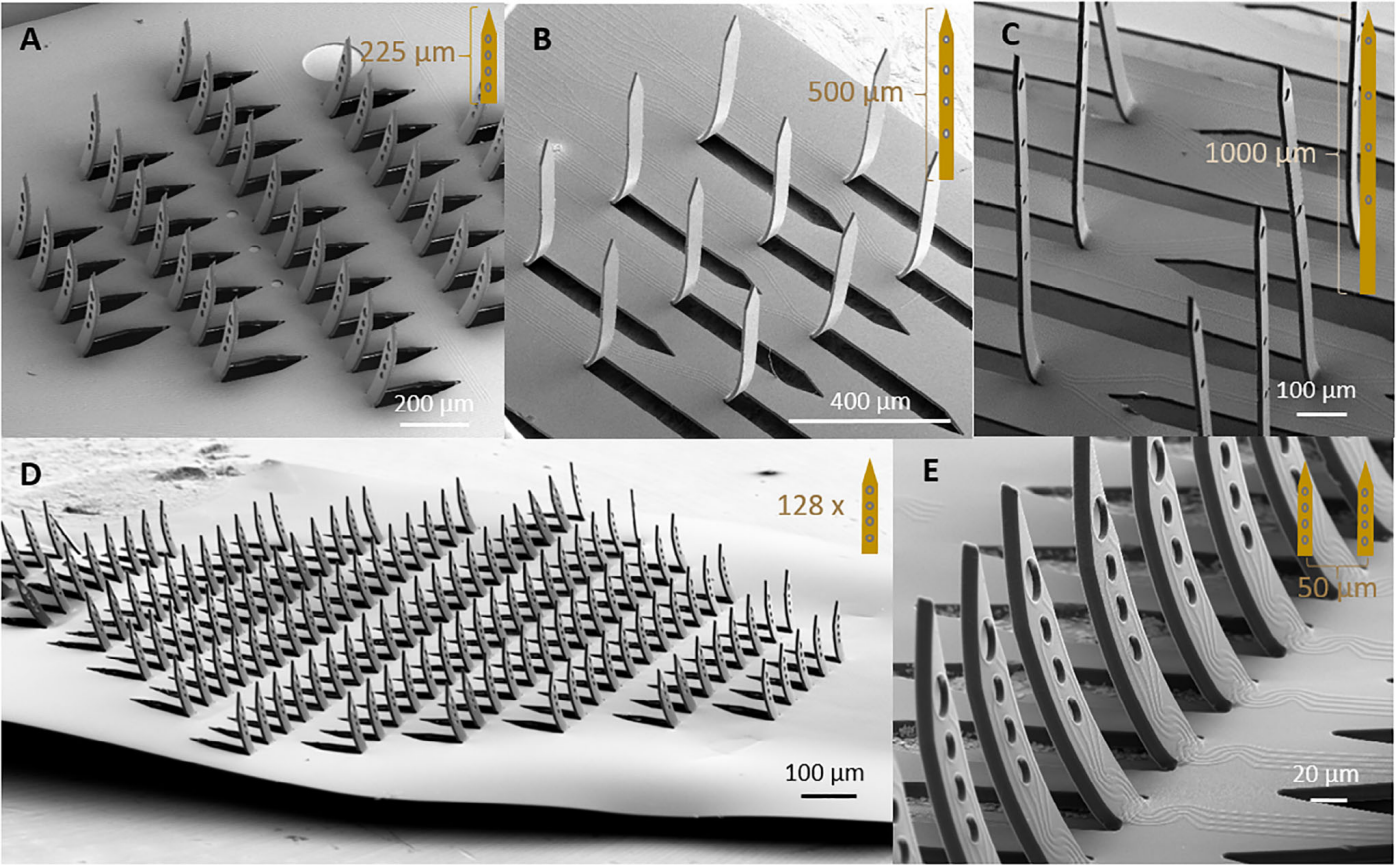
Variants of the 3D *kirigami* MEA design. Depending on the intended application, individual shank heights are feasible: A) from 225 µm for the retina and brain slices, B) over 500 µm for the cortex of rodents, C) to so far up to 1000 µm. D) Exemplary 3D *kirigami* MEA with up to 128 shanks on one flexible probe and E) an intershank distance as low as 50 µm.

Our *kirigami*‐based process allows the facile customization of 3D flexible MEAs with different laminar and spatial requirements, thus enabling the implementation of designs that meet the spatial resolution for a large range of different neural applications. For example, 3D *kirigami* MEAs with shank lengths up to 225 µm (*Kiri*‐225, **Figure** **2**A) are ideal for recording from within the laminar structure of neural slices, such as explanted retinas or brain slices, while larger shank lengths up to 500 µm (*Kiri*‐500, Figure 2B) or 1 mm (*Kiri*‐1000, Figure 2C) provide access to deeper cortical layers in the intact rodent brain. While shanks longer than 1 mm were not tested, self‐buckling calculations revealed that when maintaining the same cross‐section of the PaC shank, a critical length of *L*_c_ = 20.6 mm is theoretically feasible. Hence, to enable intraneural access, each shank contained multiple electrode sites, and to interact with the surface of nervous tissues, we incorporated surface electrodes on the planar surface of the 2D *kirigami* template.

Additionally, the protruding structures of both the lower and upper molds must be mechanically stable to avoid self‐buckling if straight shanks are desired. This stability depends on the geometry and dimensions of the protruding structures, as well as the mechanical properties of the mold materials and the lithographic techniques employed. In this work, we used two‐photon lithography to enable the 3D printing of structures with aspect ratios (shank length/width) of up to 16.3. Calculations for self‐buckling in the mold designs indicated that the *L*_c_ for the protruding blocks is at least 61.9 mm. This exceeds the maximum printing range of the tool used (max. height: 8 mm), suggesting a potential limit to producing longer shanks. Furthermore, given that the molds can be fabricated with any additive manufacturing process that allows µm–mm resolution (e.g., digital light processing printers or projection micro‐stereolithography^[^ ^39^ ^]^), and that 3D designs are derived from 2D templates, customizability in the design of the array of shanks is an advantage. In this work, we tested shank arrays with parallel rows and diamond‐shaped designs (Figure S3, Supporting Information). Irrespective of the design, the shanks are folded in an upright position with a bending angle of ≈90°, as confirmed by the processing of scanning electron microscopy (SEM) images with upright shanks oriented laterally to facilitate angle measurements. For instance, the bending angle of the *Kiri*‐225 probes yielded an average of 89.8° ± 1.2° (*N* = 5 shanks), while for the *Kiri*‐500, it resulted in an average of 86.9° ± 2.7° (*N* = 10 shanks) (Figure S3C, Supporting Information).

Exploring the shank folding scalability of the matched die forming process, devices with up to 128 shanks, each 50 µm wide and 225 µm long and an intershank spacing of 100 µm were successfully fabricated (Figure 2D). To increase the electrode count, each shank contained four electrodes for a total of 512 electrodes. Two strategies would allow further increasing the electrode count without increasing processing time. The first strategy involves reducing the width of the feedlines and the diameter of electrodes, implying a geometrical constraint given by the resolution limits of the lithographic methods used. By optimizing the lithography process, it is possible to calculate the number of feedlines and electrodes that fit not only within a single shank but also the available area in the 2D *kirigami* template, while also maintaining a three‐layered sandwich with a single metal stack layer. In our case, feedlines down to 3 µm and electrodes up to 25 µm were implemented with maskless lithography. Nonetheless, submicron features are also achievable with other techniques, such as electron‐beam lithography as reported by others.^[^ ^40^ ^]^

A second strategy consists of increasing the shank density; however, the success of the mechanical pressing is limited by the remaining solid area in the 2D *kirigami* template, especially the inter‐shank spacing. The solid areas serve as mechanical support during the matched die compression. Therefore, the closer the shanks, the smaller the inter‐shank area becomes, and the higher the compressive stresses, leading to structural instability by ripping the 2D template (Figure S4, Supporting Information). To further explore this limitation, a *Kiri*‐225 probe with an intershank spacing of 50 µm was successfully folded (Figure 2E). A reduction in intershank spacing resulted in a shank density from 28 shanks mm^−2^ to 37 shanks mm^−2^ in *Kiri*‐225 probes. For longer shanks, the remaining solid area is further reduced. Consequently, increasing the shank height imposes constraints on shank density, yielding 18 shanks mm^−2^ for the *Kiri*‐500, while for the *Kiri*‐1000, a shank density of 10 shanks mm^−2^ was already within the fabrication limits as discussed.

Considering the design requirements described above, our *kirigami* folding process showed a successful folding yield of 98% (*N* = 100 devices including all designs). Successful folding was defined as the probe being separated from the molds without any difficulties (e.g., without sticking to the mold), and all shanks being folded upright. After thermoforming, the 3D *kirigami* MEAs were coated with poly(3,4‐ethylenedioxythiophene:poly(4‐styrenesulfonate) (PEDOT:PSS) via chronoamperometry to enhance the electrochemical properties of the base Au electrodes. After electrodeposition, we achieved a yield of 47% ± 16% for successful PEDOT:PSS deposition in different runs of the 2D fabrication process (*N* = 1721 electrodes distributed on 100 probes, and 7 fabrication runs). The high standard deviation and optical inspection indicate that the success of the process depends heavily on the accurate handling of the 2D *kirigami* template as well as on the quality of the underlying metal layer. Although the folding process has the potential to be carried out in an automated manner with, e.g., pick and place techniques and the use of micromanipulators, the alignment of the 2D *kirigami* template with the corresponding molds and the folding of the probes in this work were carried out manually. Hence, a learning curve was observed as the yield was lower in the first fabrication compared to the last run. When handling the 2D *kirigami* template during folding, hectic movements during the alignment can lead to cracks in the PaC layer, which can extend to the metal layer. Considering this learning curve, we achieved a yield of 80% (*N* = 271 electrodes, last fabrication run) after the electrodeposition process.

### 2.3 Performance of 3D *Kirigami* MEAs

#### 2.3.1 Mechanical Considerations

**Figure 3:**
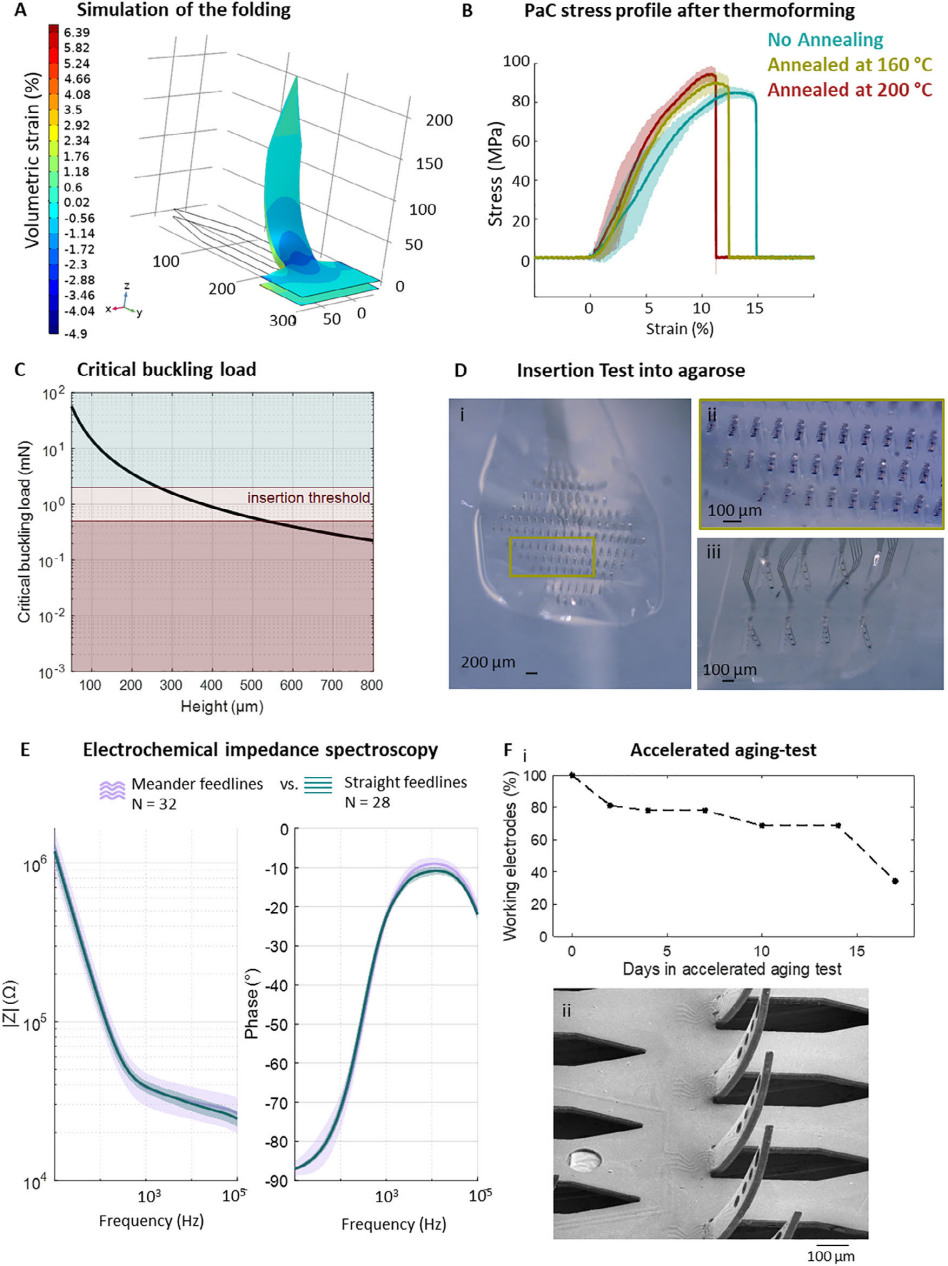
Mechanical and electrochemical performance of 3D *kirigami* MEAs. A) FEM analysis of the volumetric strain upon folding a flexible PaC shank at a 90° angle. B) Stress–strain (time) curve of 10 µm PaC films untreated (blue, *N* = 7), annealed at 160 °C (green, *N* = 8) and annealed at 200 °C (red, *N* = 8). C) Theoretical buckling load of a 10 µm thick and 50 µm wide PaC slender column of different heights. The insertion threshold according to the literature (0.5 to 2 mN) is marked in red. D) Insertion of a *Kiri*‐225 with 128 shanks (D_i_) and zoom‐in (D_ii_), and insertion of a *Kiri*‐500 with eight shanks into tissue phantom (agarose gel) (D_iii_). E) Bode plot of the electrochemical impedance spectra of electrodes with a diameter of 15 µm located in PaC shanks with meander or straight feedlines at the kink (mean ± standard deviation). F) Accelerated aging‐test of *Kiri‐225* probes. The working electrodes (in %) were determined on each measuring day using electrochemical impedance spectroscopy (E_i_) (*N* = 38 electrodes across three probes) SEM image of *Kiri*‐225 after 45 days in 60 °C immersed in phosphate‐buffered solution (1× PBS) (E_ii_).

When folding the 2D *kirigami* template, the PaC–metal–PaC sandwich that composes the implant is exposed to mechanical stress. When a shank bends upward, the top surface compresses while the lower surface stretches (**Figure** **3**A). Accordingly, finite element method simulations in COMSOL showed a maximum of −2.3% volumetric strain at the center of the kink region on the top side and 1.2% on the bottom side. After mechanical testing, we determined the Young's modulus of the 10 µm‐thick PaC layer thermoformed at 160 °C to be 1.70 ± 0.32 GPa. Consequently, we reached a strain of up to 13.93% ± 11.25% at break. Despite the high standard deviation in the measurements, a maximum strain of 2.3% during folding does not mechanically alter the PaC layer as it is still within the linear (elastic) region of the material. Hence, assuming a homogenous volumetric strain distribution, the metal layer at the middle of the two 5 µm‐thick PaC layers is exposed to 0.55% strain, which is not damaging the Au.^[^ ^41^ ^]^ After folding, SEM inspection confirmed a PaC–metal–PaC sandwich structure with PaC wrinkling at the kink area of the shanks (Figure 2). This observation supports the assumption that the feedlines (straight as well as meander‐shaped ones) were not damaged during the folding process.

To determine an optimal thermoforming protocol, we analyzed the mechanical properties of flexible PaC exposed to thermoforming processes using maximum temperatures of 160 or 200 °C and compared the results to untreated samples (Figure 3B; Table S1, Supporting Information). For 200 °C the Young's modulus and stress and strain at break are statistically different in comparison to untreated PaC stripes. In turn, thermoforming at 160 °C does not significantly change the Young's modulus. Thus, we selected 160 °C as the optimal thermoforming temperature. Given its Young's modulus, PaC could be considered a stiff material in comparison to the soft and viscoelastic nature of most neural structures.^[^ ^42^ ^]^ However, PaC facilitates the cross‐sectional optimization of the penetrating shanks, thereby minimizing not only its bending stiffness (Table S1, Figure S5, Supporting Information) but also its volumetric footprint upon implantation. This is particularly salient in scenarios where softer materials, such as silicone rubbers or viscoelastic polymers,^[^ ^43^ ^]^ were to be considered, as the processing challenges associated with these materials at miniaturized dimensions often result in a thickness at least ten times greater than the dimensions proposed in this work.

Additionally, we evaluated the mechanical stability of the flexible 3D *kirigami* MEAs by predicting the critical buckling load of individual shanks to assess their penetration capability into neural tissue. To penetrate neural tissue, an insertion force *F*_i_ between 0.5 and 2 mN is needed.^[^ ^42^ ^]^ If *F*_i_ exceeds the critical buckling load of the shanks, they fail due to bending upon tissue penetration. Thus, we predicted the insertion likelihood by calculating the Euler's critical buckling load of a 10 µm‐thick PaC layer stack thermoformed at 160 °C (Figure 3C). This showed that a maximal insertion force of 0.5 mN enables the insertion of shanks with a length up to 530 µm into neural tissues, e.g., the brain without meningeal layers such as the dura, without using an additional insertion aid.

The implementation of shanks that exceed 530 µm necessitates the optimization of the shank cross‐section. One potential approach involves augmenting the thickness of the shank, thereby elevating the critical buckling load, and enabling unassisted insertion. Alternatively, the employment of temporary insertion shuttles, a topic that extends beyond the scope of this manuscript, could be explored for the insertion of elongated shanks. In this context, the available strategies range for example from the use of stiff shuttles (e.g., needle‐like structures or wires),^[^ ^27^, ^44^, ^45^ ^]^ the use of biodegradable polymers (e.g., PEG) to reduce the effective length of the implant,^[^ ^46^ ^]^ or the incorporation of dissolvable or mechanically adaptive stiffeners.^[^ ^47^, ^48^ ^]^ However, it is imperative to acknowledge the trade‐off between the flexible material used, the implantation complexity, and the volumetric footprint of the end probe. In this regard, cross‐sectional optimization offers advantages, especially when handling 3D probes.

Prior to functional testing in neural tissues, we evaluated the insertion performance of our *kirigami* probes with agarose tissue phantoms, demonstrating the successful insertion of a *Kiri*‐225 with 128 shanks and a *Kiri*‐500 with eight shanks (Figure 3D; Video S2, Supporting Information). In the latter case, the insertion of eight shanks resulted in a peak force of 1.1 ± 0.2 mN (*N* = 7 devices) (Figure S5B, Supporting Information), which exceeds Euler's critical buckling load of 0.57 mN for a *Kiri*‐500 shank. However, since the measured peak force reflects the combined load across all the shanks, the *F*_i_ per individual shank is expected to be lower, supporting the successful insertion predicted in Figure 3C. Additionally, during the insertion process of the *Kiri*‐500 probe, the shanks demonstrated a direct penetration of the agarose gel without any indication of bending, as evidenced in Figure 3D_iii_. As a result, *Kiri‐*500 probes were selected for later testing in vivo where longer shank lengths were required.

#### 2.3.2 Electrochemical Characterization

A PEDOT:PSS coating was electrochemically deposited on the Au electrodes to improve the electrochemical performance of the 3D *kirigami* MEAs. Bode plots exhibiting the electrochemical impedance of PEDOT:PSS‐coated electrodes are shown in Figure 3F and Figure S6A,B (Supporting Information). An average impedance of 39.1 ± 3.4 kΩ at 1 kHz and 1.3 ± 0.2 MΩ at 10 Hz was measured for straight feedlines for electrodes 15 µm in diameter. For meander‐shaped feedlines, an average impedance of 38.4 ± 9.0 kΩ at 1 kHz and 1.4 ± 4.1 MΩ at 10 Hz were determined respectively. While the mean impedance values show only a slight difference, the higher standard deviation in meander‐shaped feedlines suggests that the fabrication process for straight feedlines is more consistent. Therefore, straight feedlines were preferred for further experiments. The greater impedance variation in meander‐shaped feedlines is likely due to their closer proximity to the edges of the shanks, which increases their susceptibility to minor cracks, which are a direct result of the reactive ion etching (RIE) processing stage, as previously discussed.

Accordingly, the electrochemical performance of the electrodes shows the expected resistive behavior of PEDOT:PSS electrodes at the high frequency band (above 1 kHz), relevant for recording action potentials. At low frequencies (below 100 Hz), where LFPs are usually measured, the electrodes exhibit a capacitive behavior with the phase angle approaching −90°.^[^ ^49^, ^50^, ^51^ ^]^ Hence, 3D *kirigami* MEAs showed optimal characteristics for electrophysiological recordings, as low impedance values improve the recording quality of neuroelectronic interfaces.^[^ ^52^ ^]^

PEDOT:PSS electrodeposition is performed subsequent to the folding and thermoforming processing steps. Nonetheless, the impedance of folded *kirigami* MEAs showed no statistically significant difference from that of unfolded samples (43.13 ± 1.59 kΩ, *N* = 12 electrodes with a diameter of 15 µm and meander‐shaped feedlines), as determined by an unpaired *t*‐test with 95% confidence interval (Figure S6C, Supporting Information). This finding serves to further substantiate the observation that the folding and thermoforming process does not exert an impact on the electrochemical performance of the recording electrodes.

Furthermore, we investigated the long‐term stability of the PEDOT:PSS‐coated 3D *kirigami* MEAs in an accelerated aging test (Figure 3F; Figure S7, Supporting Information). Impedance measurements revealed that 80% of the electrodes remained functional within the first two days and further declined to 34% by day 17 (Figure 3F; Figure S7A,B, Supporting Information). Optical inspection confirmed that impedance degradation resulted from PEDOT:PSS layer degradation and delamination of both, Au and PEDOT:PSS electrodes, as evidenced by color changes and surface alterations (Figure S7C,D, Supporting Information). Over the 45‐day aging test, the electrode coating exhibited progressive degradation and delamination. However, no water intrusion was observed between the PaC layers nor corrugation at the feedlines, demonstrating intact passivation and structural stability and resilience of the 3D *kirigami* MEAs, whose shanks remained vertical (Figure 3F; Figure S7C_ii_, Supporting Information).

The aging‐test results indicate then that the 3D *kirigami* MEAs are suitable for short‐term experiments, exhibiting 80% functional electrodes for at least seven days in accelerated aging, equivalent to 35 days under physiological conditions. Additionally, intact passivation and structural stability were maintained for 45 days in accelerated aging, corresponding to ≈225 days (≈7.4 months in vivo). However, for long‐term chronic applications, electrode coating stability should be improved. This could be achieved by exploring alternative electrode coatings, such as PEDOT:PSS on rougher surfaces, e.g., iridium oxide or nanostructured platinum, which are known for their long‐term stability.^[^ ^53^ ^]^

Furthermore, the *kirigami* probes were used an average of four times during in vitro trials, with a maximum of 16 insertions. A slight increase in impedance was observed (see Figure S6D, Supporting Information); however, 94% of electrodes (*N* = 33) remained functional after four insertions, and 100% (*N* = 16) retained sufficiently low impedance (impedance at 1 kHz below 350 kΩ), for further electrophysiological recordings after 16 insertions. These results demonstrate the feasibility of probe reuse without compromising functionality for recording applications, and the robustness of the probes to withstand mechanical stresses due to repetitive insertions or repositioning of the probes on the neural target.

### 2.4 Applications of 3D *Kirigami* Probes

To highlight the utility of the 3D *kirigami* probes for characterizing both pathological and healthy activity across various regions in the central nervous system, and showcase their versatility in capturing neural dynamics at different spatial scales, we deployed customized versions for different applications in vitro and in vivo. We used *Kiri*‐225 probes in human brain slices to study in vitro epileptic models, and *Kiri*‐500 probes to map somatosensory and visual stimuli in acute and chronic settings in the intact brains of anesthetized and awake mice, respectively.

#### 2.4.1 Explanted Human Brain Slices

*Kiri*‐225 probes were first utilized to examine the spatiotemporal propagation of epileptic activity in acute human brain slices and organotypic cultures. The brain slices were obtained from recessed access cortical tissue of epileptic patients at the University Hospital Aachen. As previously reported, seizure‐like events (SLEs) can be triggered by pharmacological manipulation.^[^ ^54^ ^]^ An SLE is defined by three key characteristics: a paroxysmal change in neural activity that is distinct from the background activity, a temporal and spectral evolution of discharges, and a return to baseline activity.^[^ ^55^, ^56^ ^]^ During SLEs, ictal discharges (IDs) are observed, which contain Delta and Gamma waves with increased spectral power in a frequency range between 1–100 Hz, high‐frequency oscillations (HFOs) in the 250–350 Hz range, and increased multiunit activity (MUA) above 300 Hz.^[^ ^54^ ^]^ As the thickness of explanted human brain slices ranged from 250 to 300 µm, *Kiri*‐225 probes incorporating both surface and penetrating electrodes were utilized for electrophysiological recordings.

**Figure 4:**
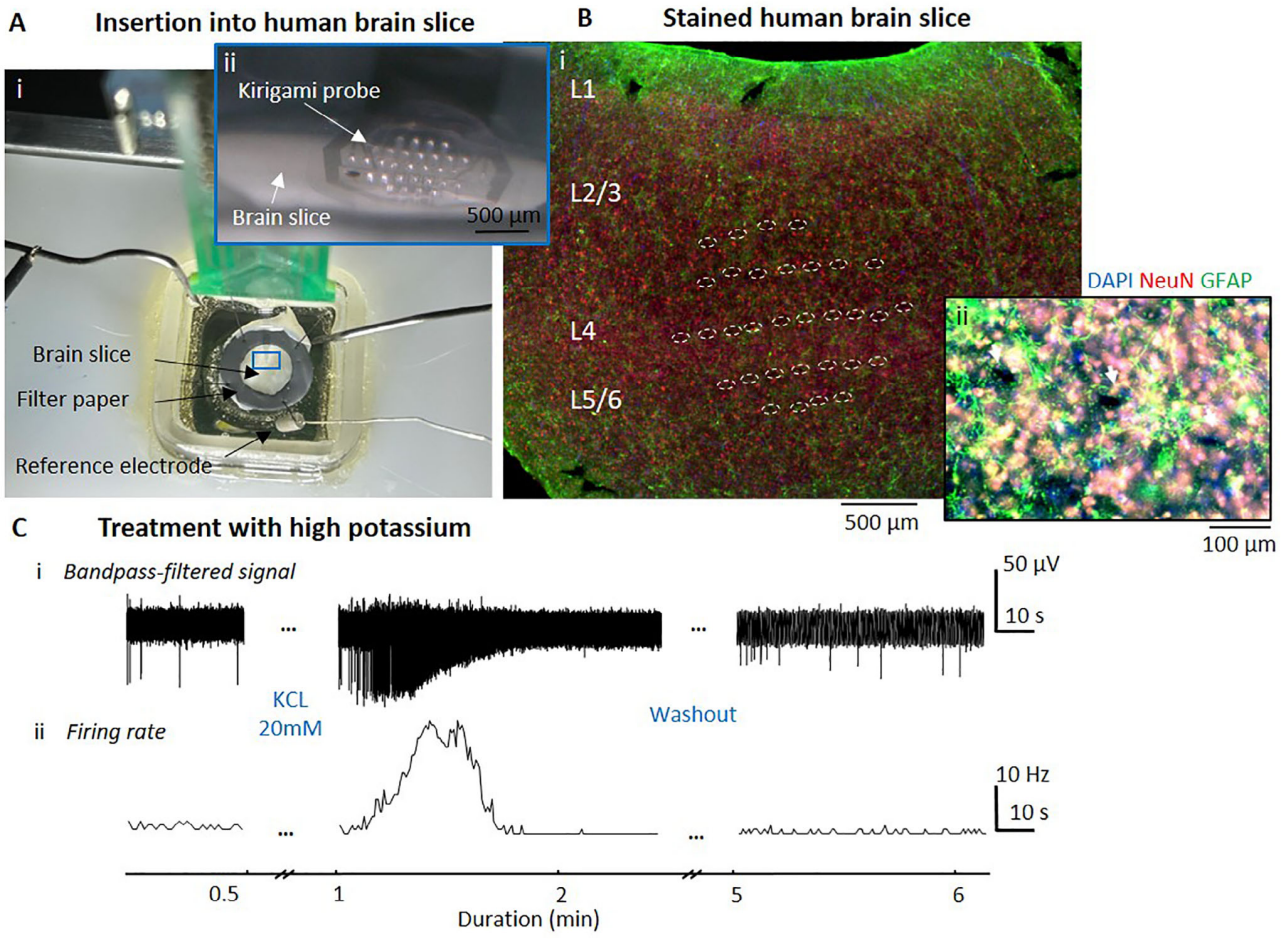
Electrophysiology with in vitro human brain slices. A) Insertion of a *kirigami* probe into a human brain slice inside a perfusion chamber with constant inflow and outflow of aCSF. The brain slice is submerged in aCSF and is supported by a doughnut‐shaped filter paper and fixed on a PDMS supporting pillow with insect pins, while the *Kiri*‐S probe is inserted perpendicular to the tissue. B) Immunohistological staining for NeuN (red), GFAP (green), and DAPI (blue) showing the insertion sites of a *Kiri*‐225 probe at a 10× (B_i_) and 20× (B_ii_) magnification. C) Human brain slice response to treatment with a high extracellular potassium concentration of 20 mm. Bandpass‐filtered data (C_i_) and the firing rate along the complete recording (bin size = 1 ms) (C_ii_), show that the cells reach a depolarization block under the treatment and go back to baseline activity after washout.

Human brain slices were submerged in artificial cerebrospinal fluid (aCSF) and placed in a perfusion chamber (**Figure** **4**A). To insert the *Kiri*‐225 probe, we first positioned the device on the brain slice surface using a micromanipulator and then gently pressed it into the tissue with a metal rod attached to a second micromanipulator (Figure S8A, Supporting Information). The probe was inserted such that individual shanks spanned multiple cortical layers. This placement was confirmed in two samples through immunohistochemical staining, which demonstrated the position of the shanks across cortical layers L2/3 to L5/6 (Figure 4B; Figure S8B, Supporting Information). This placement allowed for recordings of neural activity at different tissue depths and cortical layers. Due to the uneven surface of the brain slice and the slightly smaller surface area of the metal rod compared to the probe (see Figure S8A, Supporting Information), some shanks may not have fully penetrated the tissue. Despite this, the electrical activity captured by the electrodes connected to the data acquisition system confirmed the successful insertion of the shanks located in the middle of the probe.

We first confirmed that the electrical recordings indeed captured neural activity. To do so, we initially recorded spontaneous activity and then induced a physiological response by increasing the K^+^ concentration from 3 to 20 mm, which is known to cause a depolarization block. The latter was observed shortly after adjusting the K^+^ concentration to 20 mm, as the spiking activity strongly increased with larger spike amplitudes and higher firing rates (Figure 4C_i,ii_). Following the increase in activity, a depolarization block occurred, causing the complete cessation of subsequent action potentials. When K^+^ concentration was restored to normal levels, spontaneous activity quickly returned to its baseline state. This observation is consistent with the mechanisms of action potential formation and aligns with earlier findings.^[^ ^57^, ^58^ ^]^

**Figure 5:**
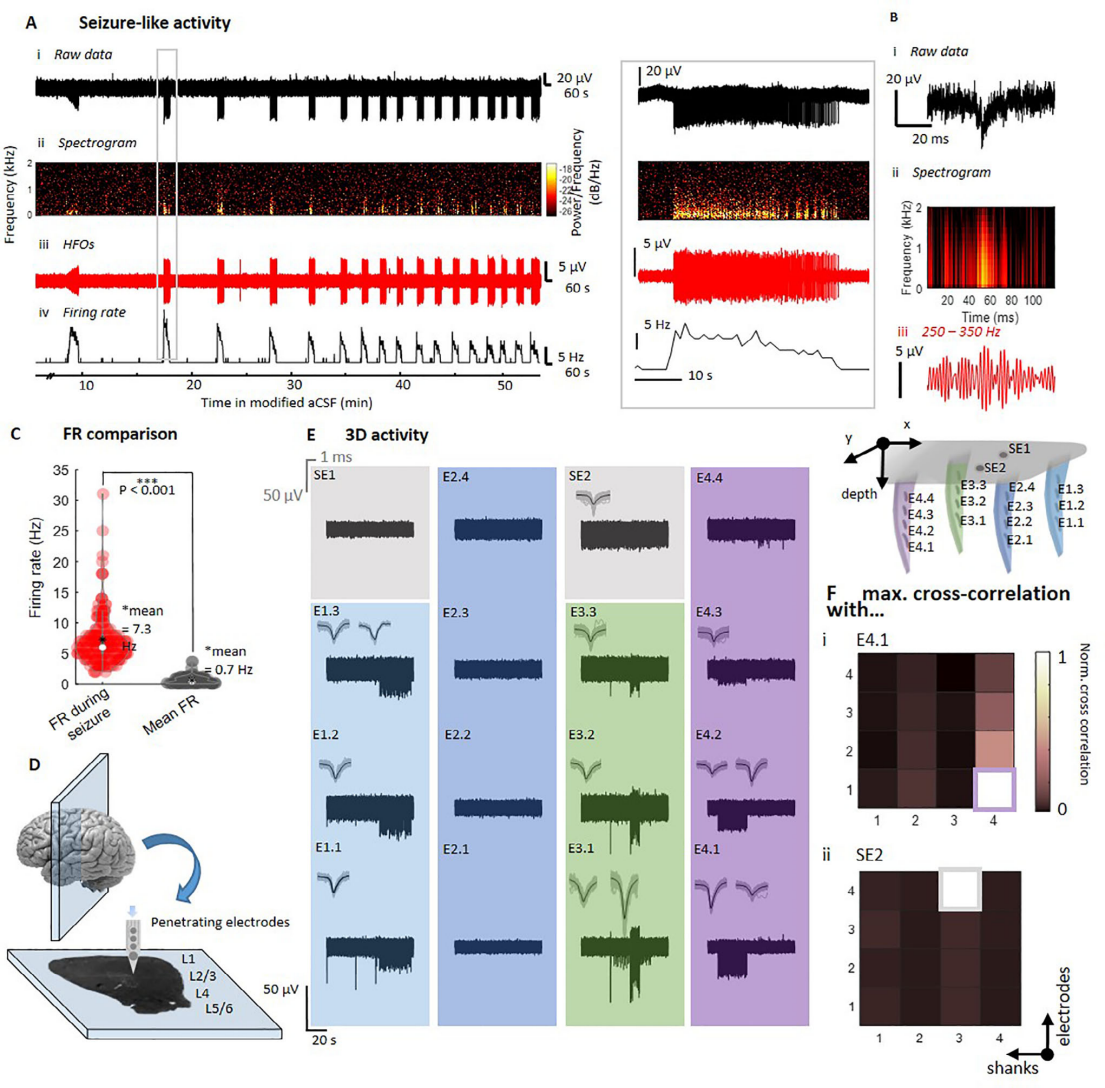
Seizure‐like activity in human brain slices. A) Electrophysiological activity upon treatment with modified aCSF (8 mm K^+^ and 0.25 mm Mg^2+^), raw data (A_i_), spectrogram (A_ii_), HFOs filtered at 250–350 Hz (A_iii_) and firing rate traces (A_iv_). B) Examples of an ictal‐like discharge, raw data (B_i_), spectrogram (B_ii_), and filtered HFOs (B_iii_). C) Comparison of firing rates during SLEs and averaged over the whole trace (*p* < 0.001 using a paired *t*‐test with *N* = 153 SLEs). D) Illustration of the placement of the shanks within the human brain slice. E) 3D map of spiking activity upon treatment with modified aCSF. The electrodes of four shanks (dark blue, light blue, green, violet) and the surface electrodes (gray) each capture SLEs at different time points. The bandpass‐filtered signal is shown in black (black scale bar) and the sorted spikes of units which fire during SLEs are shown in gray. F) Maximal value of the cross‐correlation for electrode E4.1 and SE2 with all the other electrodes. The electrode arrangement follows the pattern of (E).

In a second set of experiments, we aimed to study the spread of epileptic activity within six human brain slices by adjusting the extracellular ion concentration to induce epileptic activity patterns. Following the protocol by Pallud et al.,^[^ ^54^ ^]^ we modified the aCSF medium by increasing the K^+^ concentration to 8 mm and reducing the Mg^2+^ concentration to 0.25 mm. After introducing the modified aCSF medium, SLEs were observed. The duration of SLEs was determined by a sustained increase in the firing rate of spiking activity (100–3000 Hz) that exceeded the baseline firing rate in each individual electrode. We observed SLEs in five out of six slices from three different patients with a signal‐to‐noise ratio (SNR) of up to 200.9 and mean of 6.0 ± 1.6. On average, an increased firing rate during SLEs lasted 51.4 s, with a standard deviation of 73.5 s (*N* = 88 SLEs). These events occurred repeatedly, with intervals averaging 144.2 ± 170 s. Initial inter‐SLE periods were as long as 809 s but decreased over time to as short as 6 s (**Figure** **5**A). SLEs were observed after an average of 33.7 ± 13.7 min of perfusing the modified aCSF across six slices. During the SLEs, frequencies below 1 kHz predominantly contributed to the recorded electrophysiological signals, as evidenced by the increased spectral power (Figure 5A_ii_). This observation is consistent with previously reported studies.^[^ ^54^, ^55^, ^56^, ^59^ ^]^ Alongside the increased firing rates, HFOs (Figure 5A_iii_) were observed during the SLEs. Typically, the firing rate was highest at the beginning of the SLE and subsequently flattened until the end (Figure 5A_iv_). The average firing rate during an SLE was 7.8 ± 5.2 Hz, which was significantly higher than the overall average firing rate of 0.7 ± 0.7 Hz (Figure 5C). Furthermore, characteristic discharges during SLEs including HFOs were observed, exhibiting features of epileptic activity that matched temporal and spectral characteristics described in earlier studies^[^ ^54^ ^]^ (Figure 5B).

Leveraging the spatial distribution of electrodes in the *Kiri*‐225 probe, we then compared recordings from surface electrodes with those from penetrating electrodes. This analysis revealed the presence of ictal‐like discharges that were detected by electrodes located inside the neural slice, allowing for the identification of the onset of SLEs across electrodes on individual shanks. Figure 5E depicts a representative recording where the onset of an SLE (shank 4, pink column) occurred at ≈160 µm (centered on a 25 µm diameter recording electrode) below the tissue surface. The SLE spreads vertically over ≈60 µm, based on the center‐to‐center distance, between E4.1 and E4.3 but is not visible in the surface electrode. This further demonstrates the capability of penetrating 3D *kirigami* MEAs to detect SLEs within the tissue parenchyma that would otherwise be missed by surface or distant electrodes (e.g., SE2 or E4.4). Spike sorting revealed the capability of recording the spiking activity of several neurons, including multiunit waveforms and single units with a maximum SNR of 200 (average of 6 ± 3.2). The latter was more prominent during individual SLEs (Figure 5E), which exhibited average maximum spike amplitudes of 82.6 ± 9.2 µV. Furthermore, each penetrating shank captured up to two distinct units per penetrating shank during an SLE, while multiple units were identified outside the occurrence of an SLE (Figure 5E; Figure S9, Supporting Information).

Furthermore, as illustrated in Figure 5E,F and Figures S9 and S10 (Supporting Information) SLEs captured across individual shanks separated by 100 and 300 µm in *x* and *y* axis, respectively, were largely independent from each other, exhibiting distinct firing patterns (Figure 5E; Figure S10A, Supporting Information). Cross‐correlation analysis of the data from individual electrodes and all other electrodes revealed that electrodes within the same shank, such as shank 4 (Figure 5F_i_), exhibited a high degree of correlation, whereas the cross‐correlation with electrodes on neighboring shanks was low (Figure S10B, Supporting Information). Similarly, the surface electrode SE2 was not significantly correlated with any of the electrodes on the penetrating shanks, nor with the other surface electrode SE1 (Figure 5F_ii_). These findings indicate that the epileptic events captured were confined to individual shanks rather than spreading across all cortical layers. This activity pattern was observed in all the recorded slices, indicating the presence of independent local networks across shanks.

Our in vitro recordings with 3D *kirigami* MEAs demonstrate the capabilities to record brain signals with a broad frequency spectrum and spatial range, from HFOs to MUA with high spatiotemporal resolution, as evidenced by the occurrence of isolated SLEs at different time points and depths within human brain slices. These findings underscore the importance of studying neural activity patterns in brain slices not only with 2D MEAs on the tissue surface but also through 3D volumetric sampling. The slicing procedure can particularly damage cells at the tissue surface. Therefore, in contrast to traditional planar MEA systems, the penetrating *Kiri*‐225 probes provide a significant advantage by integrating both surface and penetrating electrodes. This unique configuration enables the recording of neural activity not only at the tissue surface but also within the slice parenchyma and throughout the tissue volume. The use of 3D *kirigami* MEAs in human brain slices allows for a more comprehensive investigation of pathological activity patterns by precisely localizing the spatial origin of SLEs, tracking their propagation through localized networks, and simultaneously capturing neural activity from adjacent networks. This enhanced spatial resolution offers valuable insights into the complex dynamics of neural activity in both physiological and pathological conditions.

The complexity of neural networks in human brain slices in vitro, especially in models of diseases such as epilepsy, demands advanced 3D recording devices to study the volumetric propagation of electrophysiological activity.^[^ ^60^, ^61^ ^]^ SLEs in brain slices are often artificially evoked to allow a more detailed mechanistic manipulation that is not always feasible in vivo. While rhythmic neuronal activity is often associated with epileptiform events, it is important to note that various regular brain patterns also show synchronous bursts of activity. Surprisingly, if synchrony is measured between single cells and the network (measured at the population level), this is not always highest during the epileptiform event (e.g., SLE), which reflects the complicated nature of epileptic activity.^[^ ^62^ ^]^ In this study, we could also show that SLEs in human brain slices exhibit both synchronous and asynchronous components. These events spread within the 3D structure of the slices, highlighting aspects of SLEs that might not be detectable in traditional 2D MEA recordings.^[^ ^63^ ^]^

#### 2.4.2 In Vivo Recordings from Mouse Cortex

Aside from applications in vitro, flexible 3D *kirigami* MEAs are promising tools for brain recordings in vivo, especially in the cortex. Because the design and electrode density of *kirigami* probes can be flexibly adjusted to match the spatial layout of different brain regions, this allows custom applications that require observing the spread of neural activity across different cortical layers and brain areas. Such recordings across layers and regions could be highly valuable for various clinical applications, for example to study the volumetric spread of epileptic activity throughout the cortex or for chronic BMIs to capture rich sensorimotor signals from local neural networks.

**Figure 6:**
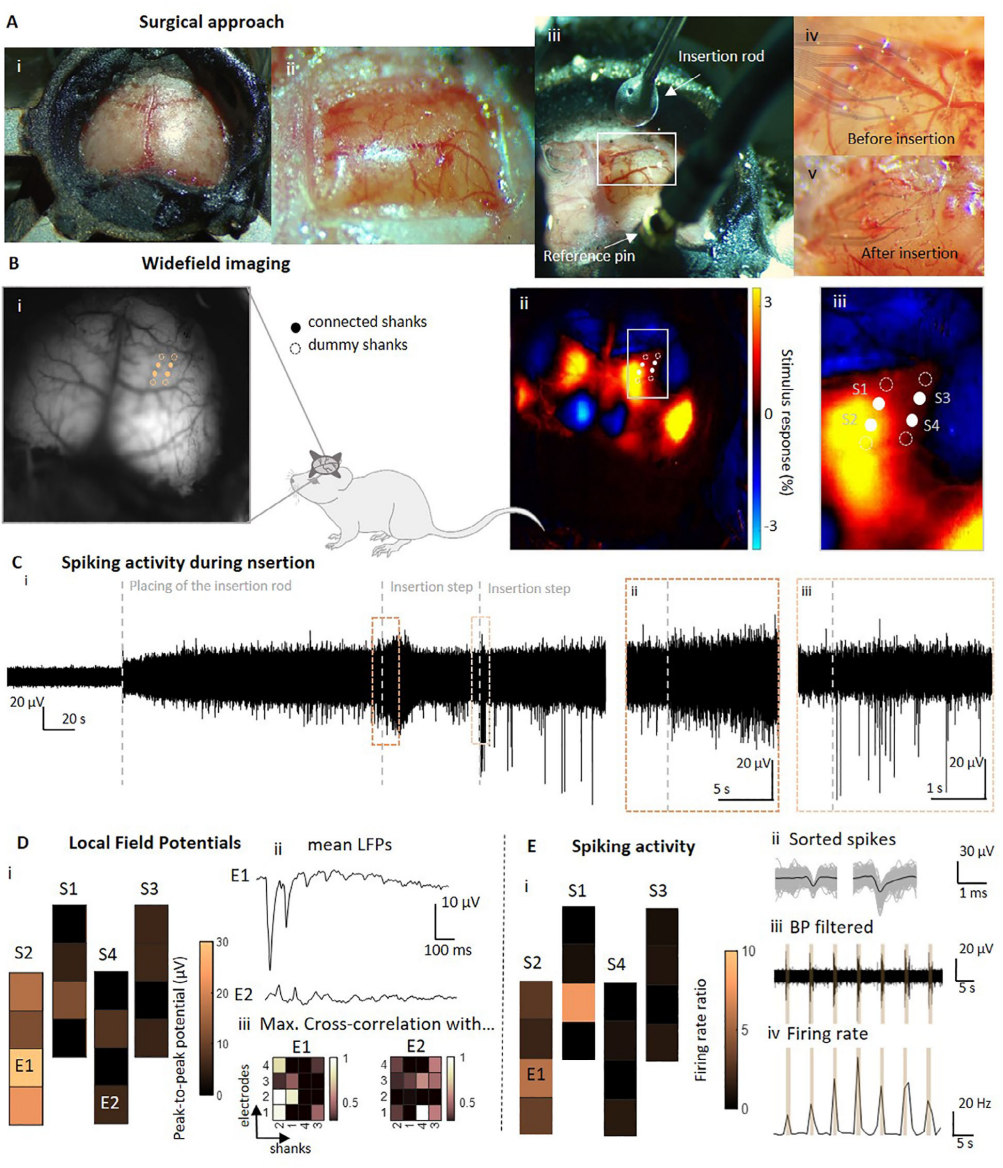
3D electrophysiological recordings of acute in vivo mouse somatosensory cortex. A) Surgical approach: a head bar is fixed on the mouse head (i) before the craniotomy (ii). The implant is pushed inside the cortex using an insertion rod (iii). The shanks are therefore placed at the desired position (iv). After the retraction of the insertion rod, they remain at their position (v). B) To identify the optimal positioning of the shanks across different brain regions (i), widefield imaging was used to identify cortical responses to tactile hindlimb stimulation (ii and zoom‐in iii). C) Exemplary intraoperative recording during insertion of *kirigami* probe. Spiking activity when placing the insertion rod on top of the implant and two following insertion steps of 200 µm each (C_i_). Gradual increase of spiking activity in the seconds after insertion (C_ii_) and abrupt increase of spiking activity directly after insertion (C_iii_). D) The peak‐to‐peak potentials were based on the averaged LFPs over 50 trials (i). Averaged LFPs show the spatial activity spread depending on the depth (z) and *x*–*y* location of the electrodes (i, ii). The heatmaps of (iii) show the maximal cross‐correlation of E1 and E2 with all other electrodes. E) Average changes in the spiking activity equally differ according to the electrodes position (i). Shown are changes in spiking activity (ii) and the respective firing rates (iii) due to tactile stimulation. The firing rates rise respectively with the stimulation (brown bars). In addition, sorted spikes are plotted for E1 (iv). The black trace shows the mean of all traces (gray).

To test the *kirigami* probes for in vivo applications, we first implanted them in the somatosensory cortex of anesthetized mice. The somatosensory cortex is composed of specialized regions for different body parts,^[^ ^64^ ^]^ which make it possible to relate their respective neural activity to tactile stimulation of the hindlimb and identify region‐ and layer‐specific functional responses (**Figure** **6**A–D). To target both superficial and deeper cortical layers in different regions, we implanted *Kiri*‐500 probes with eight or ten shanks. Before the implantation (Figure 6A), we used cortical widefield imaging to identify the hindlimb region of the primary somatosensory cortex (Figure 6B). Mice were transgenic animals that expressed the calcium indicator GCaMP6s in all excitatory neurons,^[^ ^65^ ^]^ allowing us to measure changes in neural activity as changes in fluorescence light. The somatosensory hindlimb area was then identified as the region with the strongest average neural response to brief deflections of the left hindlimb under isoflurane anesthesia (*n* = 50 trials). To obtain a 3D recording from multiple cortical regions in vivo, we then placed the shanks of the probe within the somatosensory hindlimb region, using the border of the widefield response as a guide. For this purpose, a high‐resolution vessel image (Figure 6B_i_) was used to identify the accurate location for probe placement.

Following the addition of a head bar (Figure 6A_i_), a craniotomy was placed (Figure 6A_ii_) and the dura was removed, allowing the probe shanks to be inserted at the desired position of the somatosensory cortex. Initially, a micromanipulator was employed to position the implant on the cortical surface. To facilitate the insertion of the implant into the tissue, a rod was attached to a second micromanipulator and placed directly over the implant (Figure 6A_iii_). After careful positioning, all shanks were simultaneously inserted by slowly moving the micromanipulator downward. This allowed the shanks to overcome tissue dimpling and seamlessly penetrate the cortex without any visible tissue disturbance (Video S3, Supporting Information). Prior to insertion (Figure 6A_iv_), the shanks were visible on the surface of the cortex. Following insertion (Figure 6A_v_), the insertion rod could be retracted while the shanks remained stably inserted in the tissue. The insertion of the *Kiri*‐500 probe into the mouse cortex was monitored with the intraoperative recording of electrophysical signals of the brain, ensuring the precise positioning and penetration of all shanks within the cortex (Figure 6C). The insertion process revealed a spectrum of spiking activity patterns. While some electrodes exhibited increased spiking activity in the subsequent seconds following each insertion step (Figure 6C_i,ii_), others demonstrated an abrupt elevation in spiking activity (Figure 6C_i,iii_). Following a brief interval of rest, during which tissue relaxation occurred, activity returned to its baseline state. These observations demonstrate that after insertion, physiological spontaneous activity was captured. After the insertion, we repeatedly produced tactile stimuli with a custom‐made stimulator, producing 100‐ms‐long deflections of the hindlimb with a small servomotor.

Neural responses to tactile stimulation were clearly visible as LFP deflections (signals below 100 Hz) (Figure 6D; Figure S11A, Supporting Information) and differed across the cortical locations of each shank. For each electrode, we computed the average LFP response to tactile stimulation and their peak‐to‐peak potentials. In agreement with our widefield imaging results, the neural response to tactile stimulation in the two shanks (S1 and S2) within the hindlimb region of the somatosensory cortex was much stronger compared to the lateral shanks S3 and S4 that were placed outside the hindlimb region. The strongest response occurred in electrode E1, located closest to the hindlimb region center at a depth of 300 µm, exhibiting an average potential of 28 µV, whereas electrode E2 on the neighboring shank S3 only yielded an average potential of 5.6 µV. Furthermore, correlograms revealed strong interactions across electrodes within the same cortical depth and region. This is evidenced by the high correlation observed between S1 and S2 within the hindlimb region, as well as between S3 and S4 outside the hindlimb region (Figure 6D_iii_; Figure S12, Supporting Information).

The analysis of the high‐pass filtered signals (100 to 3000 Hz) to isolate neural spiking activity revealed similar results (Figure 6E; Figure S11B, Supporting Information). During the stimulation period, the firing rates mostly increased in electrodes near neuronal units that responded most strongly to tactile stimulation. Again, the electrodes on the S1 shank, such as electrode E1, showed a strong, stimulus‐induced change in firing rates as well as high amplitude spikes, which were detected with an SNR of up to 39.5 (average of 4.8 ± 0.9), exhibiting a maximum spike amplitude of 60 µV and average maximum peak spike amplitudes of 26.7 ± 12.9 µV. This was also the case for electrodes on shank S2 whereas few changes in firing rates occurred on shanks S3 and S4. In general, spiking activity was easier to capture at deeper cortical layers, as cortical layer 1 at the surface of the cortex is mainly composed of axons. Consequently, while high amplitude LFPs were captured with all four electrodes of S1, high spiking activity was mostly captured with deeper implanted electrodes, such as E1.

**Figure 7:**
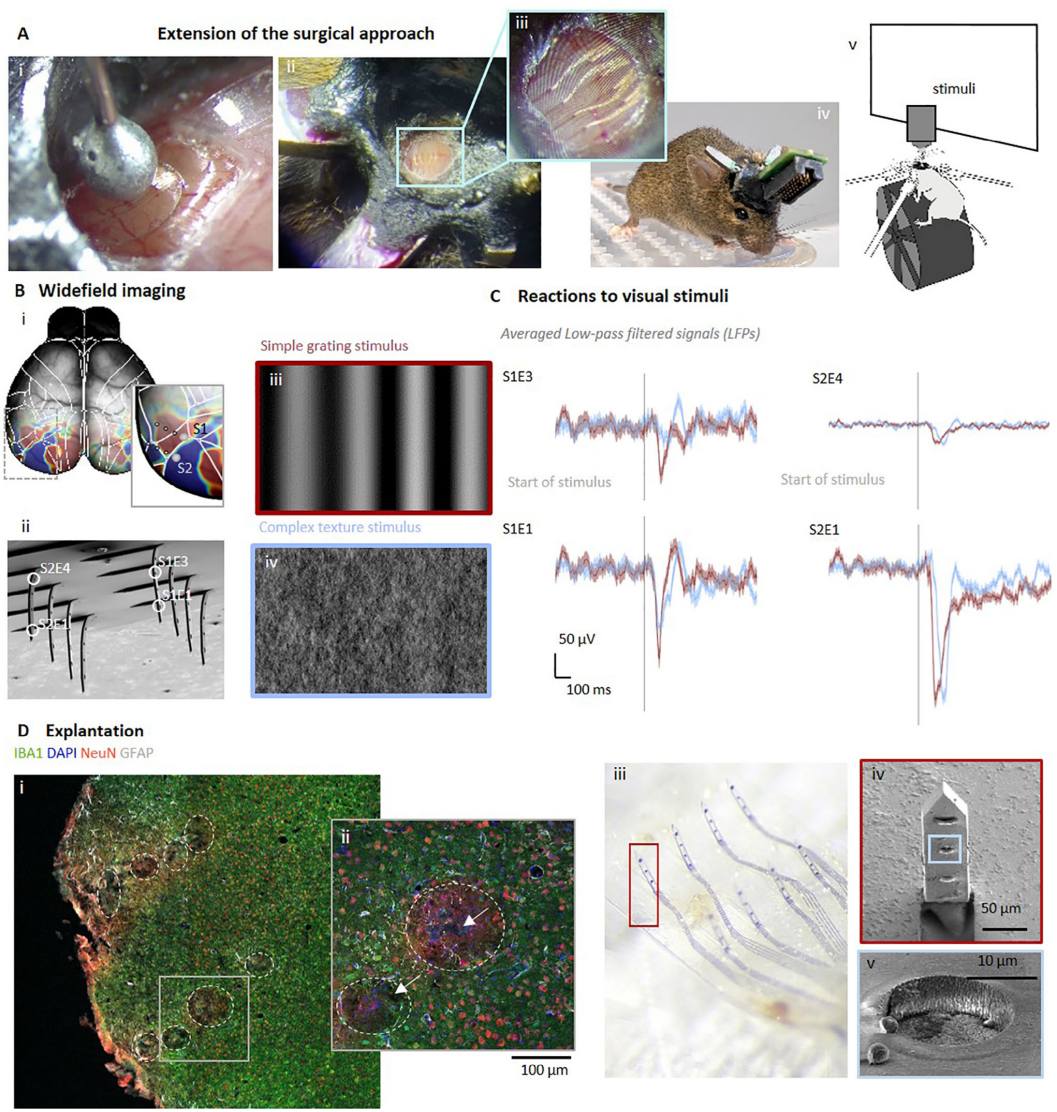
Chronic implantation and recovery recordings from the visual cortex of mice. A) For visual stimulation with awake mice, the surgical approach from Figure 6 was extended: the insertion rod to place a glass window on top of the implant (A_i_, A_ii_, and zoom‐in A_iii_). After recovery time, the mouse was woken up (A_iv_) and placed inside the experiment setup (A_v_) where it was exposed to visual stimuli. B) Widefield imaging was used to determine the desired positioning of the shanks (B_i,ii_) upon visual stimulation with simple grating (B_iii_) and complex texture (B_iv_) stimuli. C) Visually evoked averaged LFP responses in shank 1 and 2. D) Immunohistological stainings for mature microglia (IBA1, green), cell nuclei (DAPI, blue), astrocytes (GFAP, gray), and mature neurons (NeuN, red) of 20 µm‐thick horizontal slices after brain perfusion and the retraction of the implant showing implantation lesions with marked large ROI (white) at 10× magnification (D_i_). Additionally, neurons expressing GCaMP6 are also green. (D_ii_) One hole tracked through different depths and the marked large ROI (white) at 20× magnification. Implantation lesions (small ROI) match the dimensions of the shanks (white arrow pointing to ≈50 µm wide hole) and the implantation locations of the shanks. Optical inspection of the explant with light microscopy (D_iii_) and SEM (D_iv_,_v_) reveals that the shanks of the *Kiri*‐500 implant were still folded upward and adsorption of biological matter on the electrodes (D_v_).

Our results demonstrate the capability of the *Kiri*‐500 probes to record neural responses across layers and cortical regions in the living brain. To further expand upon these findings, *Kiri*‐500 probes were utilized in a chronic preparation, involving recordings made in awake mice upon recovery on the day of implantation and histological analysis one‐month postimplantation. Here, we recorded neural responses to different visual stimuli across different depths and cortical areas to characterize sensory neural responses in the awake brain. During insertion, similar to the experiment in the somatosensory cortex, we intraoperatively monitored spiking activity to position the electrodes at the desired depths (Figure S13A, Supporting Information). Distinct patterns of spiking activity emerged. Some electrodes recorded an increase in spiking activity over the subsequent seconds following insertion (as illustrated in E2.1, Figure S13B, Supporting Information), while others electrodes captured an abrupt elevation in spiking activity (as seen in E1.2, Figure S13C, Supporting Information). After a brief resting period, which lasted for ≈2 min in this example, the spontaneous activity returned to its baseline state for the majority of the electrodes (Figure S13D, Supporting Information). After the insertion, we placed a glass window on top of the implant (Figure **7**A_i_) and secured it with dental cement to the skull (Figure 7A_ii_). This allowed us to stabilize the implant in the cortex and monitor its position through the glass window (Figure 7A_iii_). For this chronic application, the front‐end connector of the implant was customized for a total form factor of 21 × 11.5 × 7.5 mm and weight of 1.6 g, ensuring that it was sufficiently small and lightweight for a mouse to carry (Figure 7A_iv_).

Awake mice were then placed inside an experimental setup (Figure 7A_v_) with a running wheel and a monitor displaying visual stimuli that fully covered the visual field of the right eye, contralateral to the implanted cortical hemisphere. To functionally determine the location of visual cortical areas before the implantation, we used widefield imaging and visual retinotopic mapping and identified the location of the primary cortex and several higher visual areas (Figure 7b_i_, blue and red regions). The functional results were in good agreement with the expected location of visual areas (white outlines), therefore we placed the *Kiri*‐500 probe to cover the primary visual cortex (V1) and several higher visual areas.

Despite efforts to minimize the weight of the front‐end connector, the interface between the flexible array and the printed circuit board of the front‐end failed one day after implantation, impeding the realization of long‐term chronic recordings. Nevertheless, we could conduct awake recordings following the recovery of the animals on the day of implantation, and additional insights of FBRs were investigated through histological examination of the tissue one‐month postimplantation (see next paragraph). In the awake recordings, we characterized how different cortical areas and layers responded to two types of visual stimulation: a simple moving grating stimulus and a complex texture (Figure 7B_iii,iv_). Focusing on LFP responses in two neighboring shanks (S1 and S2), we found clear differences in the average stimulus response across depths and locations (Figure 7B,C). Electrodes S1E1 and S2E1, positioned in the deeper cortical layers 2/3, exhibited stronger visual responses compared to the superficial electrodes S1E3 and S2E4 in layer 1. This difference in response was particularly pronounced in V1 (shank S2), whereas in the more medial anterior cortex (area AM, shank S1), the responses were more consistent across different depths. This observation was further confirmed by concomitant spike‐bursting responses to the LFPs across depths with an average SNR of 4.9 ± 0.5, and up to 17 in those electrodes where the responses were stronger, as was the case of the electrodes located in deeper layers (Figure S14, Supporting Information). Moreover, while both gratings and texture stimuli induced strong neural responses in V1, visual responses in AM were weaker and more selective for gratings over texture stimuli, especially in the superficial electrode S1E3 (red vs blue traces). Interestingly, we also observed a time shift in the response delay to visual stimuli in V1, with textures eliciting a longer delay compared to gratings. This is likely due to the increased complexity of the texture stimulus, which requires more extensive neural processing in the visual system.^[^ ^66^ ^]^ Together, these results show clear differences in the processing of complex visual stimuli across cortical depth and regions, underscoring the effectiveness of *Kiri*‐500 probes in capturing spatial dependencies both within neural regions and across cortical layers.

Four weeks after implantation, the *Kiri*‐500 probes were explanted, followed by immunohistochemical analysis of the implanted brain tissue (Figure 7D_i–iv_; Figures S7 and S8, Supporting Information) and optical inspection of the explanted devices (Figure 7D_ii_). 20 µm‐thick horizontal sections of the cortex were stained for mature neurons (NeuN, red), cell nuclei (DAPI, blue), astrocytes (GFAP, gray), and microglia (IBA1, green). The implantation footprint of the shanks is visible as small lesions (dark holes) referred to as small ROIs. Additionally, the slices reveal FBRs in tissue regions in proximity to these small ROIs (Figure 7D_i_,_ii_, white circles). This FBR is evident as fluorescent changes in the surrounding tissue (large ROI), with small lesions (small ROIs) typically located midway and occasionally off‐center within the affected areas (Figure 7D_ii_, white arrows). The insertion footprint, which contains multiple footprints of individual shanks, could be tracked throughout a depth of 260 µm. The mean area of the small ROIs, which was 478 ± 380 µm^2^ (*N* = 24), correlates with the dimensions of the shanks. The mean size of the affected regions (large ROIs) exhibiting an FBR was 7467 ± 373 µm^2^ (*N* = 56 large ROIs across 11 slices), which is ≈15 times the cross‐sectional area of the shanks (500 µm^2^). The relative size of the affected region is within the same range of observed FBRs in studies employing flexible single‐shank polymer devices (large ROIs ≈7–39 times the cross‐section of the shank^[^ ^67^ ^]^). However, in these studies, stiff shuttles were utilized for the implantation, resulting in larger footprints (small ROIs ≈2–8 times the cross‐section of the shank^[^ ^45^, ^67^ ^]^) than those left by individual shanks of the 3D *kirigami* probes.

Examination of the larger ROIs (FBR) across various depths was discernible until Z13, corresponding to an approximate depth of 260 µm, and exhibited a trend of larger footprints in superficial layers (layer 1) and smaller in deeper layers (layers 2/3) (Figures S15 and S16A, Supporting Information). The latter is also consistent with straight insertion footprint traces observed down to ≈300 µm inside the cortical parenchyma in coronal slices upon the acute insertion of DiI‐coated *Kiri*‐500 shanks (Figure S17, Supporting Information). In contrast, the smaller ROIs were only visible until slice 10, corresponding to an insertion depth of ≈200 µm (Figures S15 and S16B, Supporting Information). This trend is consistent with previous observations^[^ ^68^ ^]^ and can be explained by the fact that the shanks have a reduced cross‐sectional area toward the tip, resulting in a smaller or even indiscernible footprint in the deeper layers. Figure 7D_ii_ shows the affected insertion area on slice 8, corresponding to a depth of 160 µm, with a reduction in astrocyte density near the insertion area (small ROIs). This was also demonstrated by analyzing the relative fluorescence intensity (Figure S16D, Supporting Information), which also indicated a notable reduction in the number of astrocytes observed in the large ROIs (Figure S16C, Supporting Information), similarly to previously documented studies.^[^ ^69^ ^]^ In contrast, nuclei and neurons were not observed directly at the implantation site (Figures 7D_i_ and 7D_ii_). Similar observations were documented in other studies.^[^ ^45^ ^]^ The expression of the GCaMP indicator in excitatory neurons of the genetically modified mice, which also appear green, interfered with the ability to reliably analyze IBA1 staining.

However, it should be noted that the slicing was likely not perfectly perpendicular to the insertion direction, as achieving perfect alignment is very challenging. The total insertion depth may therefore have been underestimated, and the size of the ROIs overestimated when the slices were not cut perpendicular to the shank length. Additionally, pulling during implant retrieval caused irregularly shaped insertion holes, leading to considerable variability in the measured footprint areas (both large and small ROIs). Consequently, the assessment of FBRs is likely overestimated due to both the retraction of the implants and the slicing process.

Nonetheless, the shanks of the probe remained accurately folded upon implant retrieval (Figure 7D_iv_,_v_), and SEM inspection of the electrodes showed no evidence of delamination aside from the expected minor adsorption of biological matter. While these findings are encouraging, it is important to note that the possibility of electrode coating failure on the electrodes located on individual shanks cannot be completely ruled out. This is because their upright position during the SEM inspection process made it difficult to conduct a thorough optical examination of all electrodes. Nonetheless, this demonstrates the long‐term stability of the *kirigami* folding (Figure 7D_iii–v_). Together, these experiments demonstrate that our 3D *kirigami* probes can record neural activity from multiple depths and brain regions in both anesthetized and awake mice, enabling a detailed investigation of neural response patterns. Moreover, although stable packaging solutions are still required for chronic recordings, our findings show that the *kirigami* structure of the implant is capable of withstanding both acute and chronic in vivo implantation, with minimal astrocyte accumulation, a key indicator of FBRs, especially in the deeper cortical layers.

## 3 Conclusion and Outlook

This work introduces a novel, scalable *kirigami*‐based method for fabricating flexible 3D MEAs with both surface and penetrating electrodes. Our matched die compression folding technique enables serial manufacturing, allowing the simultaneous formation of up to 128 shanks. This approach facilitates the microfabrication of high‐density 3D MEAs with adaptable configurations for diverse neural applications. For instance, shanks measure 225 µm suit in vitro studies with human brain slices, while 500 µm shanks target cortical recordings in mice.

Our *kirigami* MEAs surpass existing flexible 3D neural implants (Table S2, Supporting Information), achieving shank lengths up to 1000 µm, intershank spacings as small as 50 µm, and electrode counts up to 512. Although fabrication remains manual, it yields high reliability and rapid production. While we demonstrated arrays with up to 512 electrodes, only 16 or 32 electrodes were connected due to current connector limitations. The primary challenge lies in developing a compact front‐end connector that accommodates all electrode wires, as increasing electrode count necessitates larger connectors. Potential solutions, such as multiple connectors (e.g., Omnetics or slim stack), result in bulky designs unsuitable for mice. An optimal solution would be a wireless, high‐channel connector to facilitate long‐term recordings. However, to the best of our knowledge, no commercial wireless front‐end currently supports 512 channels.

Our 3D *kirigami* MEAs feature a small cross‐sectional footprint and flexible, biocompatible materials with low bending stiffness, ensuring mechanical stability for a shuttle‐free insertion and optimal tissue integration. Electrochemical impedance spectroscopy further confirmed low electrode impedances across the devices, facilitating high‐SNR recordings of neural activity. However, accessing deeper cortical layers requires adjusting the shank's cross‐sectional dimensions to increase its critical buckling load, which can be achieved by increasing its width or thickness.

3D *kirigami* MEAs enable the study of spatial dependencies in electrophysiological activity in both in vitro and in vivo applications. In complex neural systems, such as the cortex in diseased conditions like epilepsy, high spatial and temporal resolution is crucial for improving diagnostics and treatments. Our study shows how flexible 3D *kirigami* MEAs enable the recording of a broad range of physiological brain signals, including LFPs (<100 Hz) and action potentials (100 Hz–3 kHz), as well as pathological oscillations, such as HFOs (250–350 Hz), in an induced epileptic model in human brain slices. Along with the spatial arrangement of the electrodes, 3D *kirigami* MEAs facilitate the study of spatial dependencies in electrophysiological activity patterns in both in vitro and in vivo applications.

After four weeks of implantation, the *kirigami* shanks remained structurally intact, with no unfolding or evident signs of electrode coating delamination, demonstrating structural and material stability. While implant packaging requires further optimization to deploy the full potential of the *kirigami* probes in chronic applications, we observed a mild insertion impact, likely due to the flexible and biocompatible properties of the *kirigami* implant. Nevertheless, it is imperative to underscore the necessity of a comprehensive chronic study on FBRs employing optimized histological methods for 3D neural implants to ensure a thorough assessment of tissue damage.

From in vitro human brain slices to the in vivo cortex of mice, our experiments underscore the complexity of neural networks, particularly in diseased models where 3D recording platforms are a crucial tool to capture electrophysiological activity patterns across layers and brain regions. Our approach enables the straightforward fabrication of flexible 3D MEAs, capable of sampling the 3D architecture of neural tissues with highly customizable designs. This innovation combines the spatial sampling and resolution of devices such as the Utah and the Michigan array into a single, flexible neuroelectronic interface. With the ultimate goal in mind to better understand and consequently treat neurological disorders, such as epilepsy or loss of sensory functions, the flexible 3D *kirigami* MEAs presented in this work offer a highly valuable tool for future investigation.

## 4 Experimental Section

### 4.1 Fabrication of *Kirigami* 3D Implants: 4.1.1 Fabrication of 2D *Kirigami* Template

The fabrication of the 2D *kirigami* template comprised the interleaved deposition of two flexible thin film layers, one metal layer, and an electrode coating. All the steps were conducted in a certified cleanroom environment to ensure a stable fabrication.^[^ ^70^ ^]^

First, a 5 µm‐thick PaC layer was deposited on a host silicon wafer via chemical vapor deposition using a PDS 2010 Labcoater 2 (Specialty Coating Systems Inc., USA). In a second step, a metallization process was performed using a lift‐off technique. Here, the metal base layer for contact pads, feedlines, and electrodes was patterned after spin‐coating the negative photoresist AZ LNR‐003 (MicroChemicals GmbH, Germany) at 4000 rpm for 45 s with a ramp of 500 rpm s^−1^, followed by a soft‐bake at 120 °C for 2 min on a direct contact hot plate. The photoresist was then exposed at 320 mJ cm^−2^ with a defoc of 2 and a CDB (critical dimension bias) of 800 with UV at 375 nm using a maskless aligner (MLA 150, Heidelberg Instruments, Germany), followed by a postexposure bake at 100 °C for 90 s on a direct contact hot plate, a developing step in AZ 326 MIF (MicroChemicals GmbH, Germany) for 90 s, and a cleaning step in deionized water. The wafer was then evaporated with a metal stack of 20/100/10 nm of Ti/Au/Ti using an electron‐beam assisted evaporation machine (Balzer PLS 570, Pfeiffer, Germany). Afterward, a lift‐off process was conducted in an acetone bath for 2.5 h to wash off the sacrificial material and photoresist. Then, a second flexible PaC passivation layer with a thickness of 5 µm was deposited as described before. Next, the flexible polymer was removed from the 3D cut‐outs of the *kirigami* design, as well as the outline of the shape of the device, the contact pads, and electrode openings. Here, an etch mask was patterned on top of the last PaC layer by spin‐coating the photoresist AZ 12XT (MicroChemicals GmbH, Germany) at 1000 rpm for 180 s with a ramp of 200 rpm s^−1^, performing a soft bake using a hot plate at 110 °C for 4 min, and exposing it to a dose of 350 mJ cm^−2^, a defoc of 2, and a CDB of −800 with UV at 375 nm with a maskless aligner. Afterward, the wafer was subjected to a postexposure bake using a hot plate at 90 °C for 1 min, followed by a developing step of 2 min using AZ 326 MIF. After patterning the etch mask, an RIE step using an O2/CF4 gas mixture was conducted to etch PaC. A second RIE step was performed to etch the top 10 nm Ti‐layer using an O2/Ar gas mixture. After RIE, the etch mask was stripped using AZ 100 remover (MicroChemicals GmbH, Germany) and then rinsed in isopropanol.

The 2D *kirigami* templates were then released from the host silicon wafer using droplets of water and tweezers. The 2D *kirigami* device was then flip‐chip bonded on top of a customized PCB. First, the PCB was preheated at 180 °C on a direct contact hot plate and the low‐temperature solder alloy Sn42/Bi58 (AMTECH, USA) was applied on the contact pads of the board, which allowed the formation of liquid bumps on each contact pad. Lowering the temperature to 160 °C, the flexible probes were aligned and placed on top of the liquid solder paste bumps, which solidified after quickly removing the new chip from the hot plate and cooling it down at room temperature. The recently soldered area was sealed with a polydimethylsiloxane (PDMS) coating with a mix ratio of 1:10 and cured at 120 °C for 30 min in an oven. For all devices, 16 or 32 channels were connected.

An electrode coating to improve the electrochemical properties of the Au‐based electrodes was needed. So, a conductive polymer, PEDOT:PSS was electrodeposited. Here, a 3,4‐ethylenedioxythiophene:poly(sodium 4‐styrenesulfonate) (EDOT:PSS) solution was prepared with EDOT and PSS with a 0.1% (w/v) and 0.7% (w/v) concentrations in deionized water. Before the deposition, the probes were first subjected to electrochemical cleaning in 1× PBS at room temperature by applying 10 cyclic voltammetry cycles to all electrodes using a sweep rate of 100 mV s^−1^ and potential limits between −0.6 and 0.9 V versus a Ag/AgCl reference electrode. Then, the surface of the 3D *kirigami* device was activated with O_2_‐plasma using a pressure of 0.8 mbar and a power of 80 W for 3 min. The electrochemical polymerization of the EDOT:PSS on the Au‐based electrodes was then performed via chronoamperometry using a constant potential of 1 V for 20 s.

### 4.1.2 Fabrication of the Molds

For the fabrication of the molds, the two‐photon polymerization 3D printer Photonic Professional GT2 from NanoScribe GmbH was used. In this process, an erbium‐doped femtosecond laser source (center wavelength 780 nm) was focused into a liquid droplet of a photoresin. If the laser power exceeds a certain threshold, the photoresin will polymerize only in the focal spot of the laser and thus really complex structures with high resolution can be achieved. The molds were designed using a CAD software and converted to print job instructions using Describe (Software by NanoScribe GmbH). A Zeiss 25X NA0.8 objective was used and IP‐S (NanoScribe GmbH, Germany) as photopolymer material. Using the given print recipe from NanoScribe designed for the combination of the 25× objective and IP‐S, the slicing distance was set to 1 µm and the hatching distance was set to 500 nm. A scan speed of 100 000 µm s^−1^, a Laser Power of 100%, and a Power Scaling of 1.2 lead to the best printing result with sufficient resolution and stability of the printed structure. The molds were printed onto commercial 2.5 cm × 2.5 cm ITO‐coated glass substrates (Nanoscribe GmbH, Germany) which were previously coated with 3 µm PaC, which ensured high adhesion of the print to the glass substrate. A developing step after the printing process washed away the remaining photopolymer which was not polymerized. The samples were placed into a bath of fresh mr‐Dev 600 developer for 15 min followed by another 5 min bath in refreshed mr‐Dev 600. Finally, the molds were placed into IPA for another 5 min and then air dried.

### 4.1.3 Thermoforming

After the folding of the 2D probe inside the molds, a thermoforming step was carried out to keep the 3D structure after separating the flexible probe from the molds. Researchers propose to conduct thermoforming in a vacuum oven to prevent thermal oxidative degradation.^[^ ^41^ ^]^ Additionally, a slow temperature ramp is required in the heating and cooling step to prevent failure of the thermoplastic material due to thermal and mechanical stresses. When optimizing a thermoforming protocol, temperature is a more critical factor than time, as it has been observed that the crystallization reaction is completed during the first minutes of the thermoforming process.^[^ ^71^ ^]^ It is important for the thermoplastic material to have a melting point lower than the glass transition temperature of the material of the mold. Failure can occur due to sticking of the PaC to the molds in case of higher temperatures (>170 °C). On the other hand, if the temperature is too low or the process too short, the shanks fold back down again. Therefore, it is required to find optimal parameters. Considering what was previously mentioned, the following thermoforming protocol is conducted.

1. The probe and the molds are placed into the oven at room temperature.
2. Temperature ramp of 5 °C min^−1^ until final temperature of 160 °C is reached.
3. The maximum temperature of 160 °C is held for 60 min.
4. The oven is turned off. Probe and molds are cold down for 120 min.
5. The probe and the molds are carefully separated.

### 4.2 Mechanical and Electrochemical Characterization

Simulations of the folding process were done using COMSOL Multiphysics and the shell module. The Young's modulus of PaC was set to 1.7 GPa, which was determined experimentally with tensile tests of PaC stripes (10 µm thickness, 2 mm widths) using the UniVert tensile tester (CellScale). For the calculation of the critical buckling load Euler's formula was used

(1) $$F=\frac{\pi^{2}EI}{{\left(KL\right)}^{2}}$$

Here, the Young's modulus (*E*) of PaC was set again to 1.7 GPa, the second moment of inertia is *I*, the column effective length factor *K* = 0.7 for a fixed‐pinned boundary condition, and *L* is the length of the shank.

The critical length for self‐buckling *L*_b_ was calculated following Greenhill's equation

(2) $$L_{b}={\left(7.83734\frac{EI}{\rho gA}\right)}^{\frac{1}{3}}$$

where *E* corresponds to the Young's modulus, *I* is the second momentum of inertia, *ρ* is the density, *g* the acceleration due to gravity, and *A* corresponds to the cross‐section. The calculations were carried out for the protruding blocks of the lower molds as well as for a single PaC shank. In both cases, a clamped constraint for the base and a free situation at the top of the block or the shank were assumed for the boundary conditions.^[^ ^72^ ^]^ The material properties of IP‐S were given by the manufacturer (*E* = 2.1 GPa, *ρ* = 1.19 g cm^−3^ for solid structures at 20 °C). For the material properties of PaC a Young's modulus of *E* = 1.70 GPa (Table S1, Supporting Information) and a density of *ρ* = 1.289 g cm^−3^ were used.^[^ ^73^ ^]^ The cross‐section *A* was calculated at the bottom of a protruding block in the different designs used. For a rectangular block the momentum of inertia *I* was determined as follows

(3) $$I=\frac{lw^{3}}{12}$$

where *l* is the length of the protruding block and *w* is the corresponding width.

Electrochemical impedance spectroscopy was measured in 0.1 m phosphate‐buffered saline (PBS) using a Biologic VSP‐300 (Bio‐Logic SAS, Claix, France) potentiostat and a three‐electrode configuration. Here, each electrode of the 3D *kirigami* MEA was a working electrode, and a Pt wire and an Ag/AgCl pellet were used as counter and reference electrodes, respectively.

### 4.3 Accelerated Aging Tests

3D *kirigami* MEAs were subjected to an accelerated aging test, during which the devices were immersed in 1× PBS at 60 °C. The electrochemical performance was assessed via impedance spectroscopy, measured over a period of 17 days, with measurements obtained at two‐ to three‐day intervals. To complement the impedance measurements, the 3D MEAs were examined on the days of the measurements and after 45 days using an optical microscope (Keyence VK‐X100) and scanning electron microscopy (Gemini 1550 instrument (Leo/Zeiss)). The failure of the electrodes was determined by their impedance at 1 kHz with a threshold of 1 MΩ, which is equivalent to a plain Au electrode.^[^ ^49^ ^]^ The aging factor is estimated from Arrhenius law^[^ ^74^ ^]^

(4) $$\mathrm{Aging}\;\mathrm{factor}=Q_{10}^{\frac{T_{\mathrm{accelerated}}-T_{\mathrm{target}}}{10}}$$

where *Q*_10_ is a material dependent parameter, which for polymers is ≈2, *T*_accelerated_ equals 60 °C, and *T*_target_ equals 37 °C. Therefore, the aging factor corresponds to 1 survival day at 60 °C in PBS corresponds to ≈5 days at 37 °C in PBS.

### 4.4 Imaging

Pictures of 2D *kirigami* template before the folding process were taken with a Nikon L200N microscope. Pictures of the 3D printed structures were then taken using a scanning electron microscope (SEM, Gemini 1550 instrument (Leo/Zeiss)). SEM imaging was improved by enhancing the conductivity of the 3D probes by sputtering a thin layer of iridium oxide (6 nm) onto the sample (current 15 mA for 1 min). The imaging was taken at a 3 kV acceleration voltage.

### 4.5 Tissue Preparation and Surgery for In Vitro and In Vivo Recordings: 4.5.1 In Vitro Testing of Human Brain Slices

Human neocortical brain slice cultures were prepared from access tissue (cortical tissue outside the epileptic focus, resected to gain access to the pathology) obtained from patients undergoing epilepsy surgery. Approval (EK067/20) of the ethics committee of the University of Aachen as well as written informed consent was obtained from all patients, allowing spare, nonpathologic tissue, which was acquired during the surgical approach, to be included in the study. The tissue preparation was performed by the Clinic for Neurology, Uniklinik RWTH Aachen, according to published protocols.^[^ ^75^ ^]^ To ensure tissue integrity the cortex was carefully micro‐dissected and resected with only minimal use of bipolar forceps. The section is cut perpendicular to the surface; thus it contains all six layers of the cortex. It was then directly transferred into ice‐cold aCSF (in mm: 110 choline chloride, 26 NaHCO_3_, 10 d‐glucose, 11.6 Na‐ascorbate, 7 MgCl_2_, 3.1 Na‐pyruvate, 2.5 KCl, 1.25 NaH_2_PO_4_, and 0.5 CaCl_2_) equilibrated with carbogen (95% O_2_, 5% CO_2_) and immediately transported to the laboratory. During the whole process, the tissue was kept submerged in cool and carbogenated aCSF at all times. After removal of the pia, tissue chunks were trimmed perpendicular to the cortical surface and 250–350 µm thick acute slices were prepared using a Microm HM 650V vibratome (Thermo Fisher Scientific Inc). Afterward, the slices were cultured according to prepublished protocols^[^ ^75^ ^]^ or measured as acute slices. The slices were then transported to the laboratories at IBI‐3, Forschungszentrum Jülich in O_2_ saturated aCSF. Once the slices arrived, they were placed into aCSF (in mm) 124 NaCl, 24 NaHCO_3_, 3 KCl, 1.25 NaH_2_PO_4_, 1.25 MgCl_2_, 2 CaCI_2_, and 10 glucoses oxygenated with carbogen and placed on a hot plate. For electrophysiological recordings, the brain slices were placed on donut‐shaped filter papers, fixed with insect pins, and placed into a perfusion chamber at room temperature. The electrophysiological recordings with human brain slices immersed in modified aCSF lasted in total 54 ± 12 min (mean ± std, *N* = 6) with the longest recording taking 77 min. Electrophysiological activity was recorded after up to 10 h in aCSF.

### 4.5.2 In Vivo Animal Testing

The mice for in vivo experiments were bred at the Institute of Biology 2 in Aachen. The experiments in this work have been approved by the Landesumweltamt für Natur, Umwelt und Verbraucherschutz Nordrhein‐Westfalen, Recklinghausen, Germany, according to German animal protection law and ethics under permit number 81‐02.04.2021.A021 and is reported according to the ARRIVE guidelines.

The mouse strains were obtained from the Jackson Laboratory and subsequently maintained at the local breeding facility at RWTH Aachen University. To facilitate the imaging of calcium‐related fluorescence, mice were generated through a double transgenic process involving the crossing of the TRE‐GCaMP6s (G6s2, JAX 024742) and Camk2α‐tTA (CaMKII‐tTA, JAX 007004) mouse strains. This process yielded reliable and consistent expression of the calcium indicator GCaMP6s across all excitatory pyramidal neurons in the cortex of adult mice. Mice were adult males between 12 and 20 weeks of age and two mice were used for acute and chronic trials, respectively.

### 4.5.2.1 Surgical Procedure

Prior to surgery, analgesia was given to the animals by subcutaneously injecting buprenorphine (0.1 mg kg^−1^) and carprofen (4 mg kg^−1^). In addition, local application of bupivacaine was used for local analgesia. During surgery, the mice were placed in a stereotactic frame with monitored body temperature (37 °C) and anesthetized using 1–5% of isoflurane. After an initial cut, the skin at the skull was gently pushed aside and the skull was cleared. Widefield imaging was initially performed with sensory stimulation to identify the ideal location for placing the *kirigami* probe across cortical areas. For low‐noise signal recordings, a reference pin was placed on the cerebellum. Then, a craniotomy of ≈4 mm in diameter was performed at the left or right hemisphere using a biopsy punch to mark the location and an orthopedic drill (Eickemeyer) to open the skull. The exposed dura was covered with PBS and cleaned. After an initial incision at the edge of the craniotomy window, a duratomy was performed with a hooked needle to gain access to the cortex. Subsequently, the *kirigami* probes were carefully placed on top of the desired position of the dried cortex using a 3‐axis micromanipulator (MTM‐3, World Precision Instruments). A metal rod, fixed on a second micromanipulator (uMp4, Sensapex), was used to push the implant to penetrate the tissue. The insertion into the cortex was done with step sizes between 100 and 250 µm and a velocity of 200 µm s^−1^. For experiments in the visual cortex, after the successful insertion of a *kirigami* probe, a glass window was placed on the implant. Subsequently, the implant was fixed on the skull using dental cement. Following the surgery, analgesia was sustained for 3 days using a combination of oral administration (buprenorphine, 0.1 mg mL^−1^ in drinking water) and subcutaneous injections (carprofen, 4 mg kg^−1^). After recovery, visual stimulation experiments were carried out on the same day of the implantation.

### 4.5.2.2 Widefield Imaging

Widefield imaging was done through the cleared intact skull, using a custom‐built setup with a tandem lens macroscope and two 85 mm objectives (Walimex Pro 85 mm f/1.4 IF; Walimex). Images were acquired with an sCMOS camera (Edge 4.2, PCO), using a python‐based software package (Labcams, https://github.com/jcouto/labcams, by Joao Couto) at a framerate of 30 Hz and a resolution of 512 × 512 pixels. Frames were acquired under alternating illumination using a blue LED (470 nm, M470L3, Thorlabs) and a violet LED (405 nm, M405L3, Thorlabs) and GCaMP6s fluorescence signals were isolated using a 525 nm emission filter (86‐963, Edmund optics) in front of the camera. Images acquired under violet illumination captured calcium‐independent fluorescence at the isobestic point of GCaMP.^[^ ^76^ ^]^ Therefore, by subtracting the linearly rescaled calcium‐independent signal from the calcium‐dependent signal acquired under blue illumination, intrinsic signal was removed due to hemodynamic fluctuations. The images were then further processed using self‐written MATLAB scripts (MATLAB R2019b, MathWorks).

### 4.5.2.3 Sensory Stimulation

Tactile stimulation to record neural responses from the somatosensory cortex during either widefield or electrophysiological recordings was performed by a custom‐built stimulator, comprised of a rod attached to a small servomotor (TGY‐306G‐HV) and controlled by a Teensy 3.6 microcontroller (PJRC LLC). By moving the motor at high speeds, the rod applied mild pressure to the hindlimb for a duration of 0.1 s every 5 s and stimulation was repeated for a total of 50 trials. To compute cortical responses to tactile stimulation during widefield imaging, the averaged cortical activity was then computed in the first 150 ms relative to tactile stimulation.

To identify the location of visual cortical regions, retinotopic mapping^[^ ^77^ ^]^ was performed during widefield imaging. Continuous periodically drifting bars with a flickering checker pattern were presented as visual stimuli. Stimuli were created using Psychtoolbox^[^ ^78^ ^]^ in MATLAB and composed of a static checker pattern with a patch size of 20° and contrast reversal at 3 Hz. This pattern was masked and only visible through a 15°‐wide bar aperture, moving over the screen with a temporal period of 0.5 Hz in all four cardinal directions in randomized order. Stimuli covered roughly 120° horizontally and 80° vertically of the visual field of the mouse and were prerendered in advance. A spherical correction was applied to compensate for distortions due to the presentation on a flat monitor. The monitor (BenQ XL2420T) was positioned at an angle of 20° to the midline and tilted 10° above the animal. The eye was positioned at the horizontal center of the monitor, 30 mm above the lower edge of the monitor and the stimulus presentation was adjusted accordingly. By combining functional responses and anatomical landmarks the imaging data were then registered to the Allen Common Coordinate Framework^[^ ^64^ ^]^ to reliably identify the location of the primary visual cortex and the surrounding higher visual areas.^[^ ^79^ ^]^

To trigger different neural responses across cortical regions either a simple moving grating stimulus or broadband random phase textures were presented to create more complex visual stimuli.^[^ ^80^ ^]^ The moving grating stimulus consisted of a full‐field horizontal grating with a spatial frequency of 0.04 cpd, moving with a temporal frequency of 1 Hz. The broadband stimulus consisted of a full‐field dense mixture of localized moving gratings with random positions, with a range of orientations between 0 and 45° and spatial frequencies between 0.004 and 0.4 cpd. All stimuli were prerendered in Matlab and presented for 5 s, followed by a 5 s interstimulus interval during which a mean gray screen was shown. Gratings and broadband stimuli were shown in randomized order for a total of 50 repetitions, respectively.

### 4.5.2.4 Sectioning of the Brain

Before perfusion, mice were anesthetized with 5% isoflurane in oxygen and given additional analgesia (Buprenorphine, 0.1 mg kg^−1^). Subsequently, an overdose of pentobarbital (150 mg kg^−1^) was given and mice were perfused with PBS after cessation of the respiratory reflex. Following perfusion of the mouse with PFA (4%), implants were retracted, and the brain was extracted from the skull. The brain was fixed for one day and then prepared for cryosectioning. To provide cryoprotection, the brains were immersed in 10% sucrose in 1× PBS overnight and then in 30% sucrose in 1× PBS until sectioning. For sectioning, the frontal lobe and cerebellum were removed, and the brain was embedded in Tissue‐Tek O.C.T. compound and frozen. The frozen brain was cut horizontally at 20 µm thickness using a LEICA CM3050 S at −23 °C chamber temperature and −21 °C object temperature. The slices were collected on Epredia SuperFrost Plus adhesion slides, dried, and then stored at −80 °C until staining.

### 4.5.2.5 Histology

Rodent and human brain slices were stained using the antibodies NeuN (MAB377, A60‐monoclonal, 4101852, MilliporeSigma, Merck) at a 1:1000 dilution; antimouse AF555 (A48287, polyclonal, WH334377, Invitrogen) at a 1:750 dilution, GFAP (AB5541, chicken, polyclonal, 3877381, MilliporeSigma, Merck) at a 1:1000 dilution; antichicken

AF647 (A32933, polyclonal, VI306861, Invitrogen) at a 1:1000 dilution, Iba‐1 (019‐19741, rabbit, polyclonal, LEJ1842, Wako Chemicals) at a 1:1000 dilution; anti rabbit AF488 (A32731, polyclonal, ZE390392, Invitrogen) at a 1:750 dilution, and DAPI (D9542, 079M4003 V, MilliporeSigma, Merck) at a concentration of 285, 5 nm. The human brain slices were washed three times in PBS, kept for 1 h in 15% sucrose, washed again three times with PBS, and then blocked for 2 h with 10% goat‐serum (with PBS‐T‐T). The samples were then incubated with the first antibodies for three days, then washed again in PBS and after that incubated overnight with the second antibodies. After doing another washing step, the slices were each stained with 250 µL DAPI and then washed again before they were immersed in fluoromount. After defrosting and drying the rodent brain slices, they were processed in a similar way: three times washing in PBS, 30 min in PBS‐T‐T, three times washing in PBS, blocking in 10% goat‐serum for 1 h, incubating with the first antibodies overnight, washed, and incubating with the second antibodies for 90 min and washed again. They were then stained with DAPI, washed and dipped in 200 µL TrueBlack, Cat: 23007, Biotium (1:20 in 70% ethanol) for 30 s. After the last washing step, they were immersed in fluoromount.

### 4.5.2.6 Data Processing of the Stained Images

Images of human brain slices were taken with an Echo Revolution fluorescence microscope (ECHO) and mouse brain slices were taken with a confocal laser scan microscope (Zeiss LSM 710) and processed using ImageJ.^[^ ^81^ ^]^ Confocal images at a 10× magnification were taken with a pixel dwell time of 1.24 µs, and laser wavelengths of 405 nm (blue), 488 (green), 561 (red), and 633 (far red) with laser powers of 2.6%, 2.2%, 1.0%, 3.0%, and a gain of 720, 780, 800, and 750, respectively. Confocal images at a 20× magnification were taken with a pixel dwell time of 0.93 µs, and laser wavelengths of 405 nm (blue), 488 (green), 561 (red), and 633 (far red) with laser powers of 1.5%, 1.0%, 1.0%, 1.0%, and a gain of 620, 720, 800, and 670, respectively. For the mouse brain slices, ROIs were selected using the wand tool in ImageJ. Then, the image was split into channels and the threshold adjusted. The watershed algorithm was used to separate overlaying cells. The particles were counted for the blue (DAPI) and red (NeuN) channel. For the fluorescence intensity the mean gray values were computed inside the ROI as well as inside a reference ROI (500 × 500 µm, placed 500 µm away from implantation site) for the relative fluorescence intensity.

### 4.6 Electrophysiological Data Acquisition and Signal Processing

For in vitro as well as for in vivo tissue recordings, the ME2100‐System (Multi Channel Systems MCS GmbH, Germany) and a 32 channel headstage (ME2100‐HS32‐M‐3m) were used. NeuroNexus adapter (ADPT‐NN‐16/32 and ADPT‐NN‐32) served to connect the headstage to the customized 16 or 32 channel PCBs. The McsMatlabDataTools Matlab toolbox^[^ ^82^ ^]^ and self‐written scripts were used to import and process offline HDF5 files created by the ME2100‐System. The raw traces were bandpass filtered with cut‐off frequencies of 100 Hz and 3 kHz using a 6th order zero‐phased Butterworth filter to extract spiking activity. For LFPs, the data were low pass filter at 100 Hz. HFOs were filtered using a 10th order Butterworth bandpass filter with cut‐off frequencies of 250 and 350 Hz. For tactile as well as visual stimulation, the LFPs were sorted into trials corresponding to the type of stimuli. Then, the responses were averaged (mean ± standard deviation) to be displayed in Figures 6D and 7C. Spike‐sorting was performed using the UltraMegaSort2000 algorithm.^[^ ^83^ ^]^ The SNR was calculated using the maximum and averaged peaks of the detected spikes divided by the standard deviation of the noise. In order to calculate the maximum value of the cross‐correlation during the SLEs in the respective electrode, the raw data trace during the SLEs of the respective electrode was cross‐correlated with all other electrodes, and the resulting data were averaged over all SLEs in the trace. This process was repeated for each electrode in Figure 5D.

### 4.7 Statistical Analysis

Statistical analysis was carried out using self‐written scripts in Matlab using the Statistical Toolbox. Data are displayed in absolute values or mean ± standard deviation if not described otherwise. *N* number of samples are noted for each data evaluation at the respective figure/table description. To compare the tensile test results of untreated versus annealed PaC stripes, an unpaired two‐sample *t*‐test with a 95% confidence interval was conducted. After confirming the normality of the data using a Lilliefors test, the data between groups were compared to verify the statistical significance of the observed patterns. A paired *t*‐test with 95% confidence interval was used to compare firing rates upon SLEs in human brain slices.

## Conflict of Interest

Forschungszentrum Jülich has filed a patent that covers the 3D *kirigami* fabrication exposed in this manuscript, listing M.J., J.A.S., L.K., A.O., and V.R.M. as inventors.

## Author Contributions

M.J. and V.R.M. conceived the development of the *kirigami* probes and planned the study. M.J. fabricated the devices with the support of J.A.S., S.D., L.K., A.O., and V.R.M. J.A.S. and S.D. fabricated the molds for the *kirigami* folding. M.J. characterized the devices with the support of S.D. and L.K. M.J. carried out FEM simulations with the support of S.D., A.O., and V.R.M. A.H. and H.K. supported the retrieval of human brain slices. M.J. carried out in vitro experiments with human brain slices with the support of S.M.P, H.K., and V.R.M. S.M. conducted the in vivo experiments in mice with the support of M.J., P.S.G., and V.R.M. P.S.G. prepared the mouse brain slices. H.K. carried out the immunohistochemical staining of human and mouse brain slices. M.J., P.S.G., and S.M.P imaged the neural slices. M.J. collected and processed data and figures with the support of S.M., H.K., A.O., and V.R.M. M.J. and V.R.M. wrote the initial draft of the manuscript. All authors reviewed and edited the manuscript. V.R.M. supervised the project.

## Supporting information



Supporting Information

Supplemental VideoS1

Supplemental VideoS2

Supplemental VideoS3

## Data Availability

The data that support the findings of this study are available from the corresponding author upon reasonable request.
