# Supplementary material for: Flexible 3D Kirigami Probes for In Vitro and In Vivo Neural Applications
Source: Adv Mater. 2025 Apr 14;37(24):2418524. doi: 10.1002/adma.202418524 (PMC12177860; doi:10.1002/adma.202418524)
Supplement: Supplementary file 1 — Supporting Information [file ADMA-37-2418524-s002.docx]

**Supplementary material**

Flexible 3D *Kirigami* Probes for *I*n *Vitro* and *In Vivo* Neural Applications

**M. Jung^1,2^, J. Abu Shihada^1,2^, S. Decke^1^, L. Koschinski^1,2,4^, P. S. Graff^1,2^, S. Maruri Pazmino^6^, Anke Höllig^5^, H. Koch^5^, S. Musall^1,3^, A. Offenhäusser^1^ and V. Rincón Montes^1*^**

^1^Bioelectronics, Institute of Biological Information Processing-3, Forschungszentrum Jülich, Jülich, Germany

^2^RWTH Aachen University, Aachen, Germany

^3^Institute for Zoology, RWTH Aachen University, Aachen, Germany

^4^Helmholtz Nano Facility (HNF), Forschungszentrum Jülich, Germany

^5^ Department of Neurosurgery, RWTH Aachen University, Aachen, Germany

^6^Department of Epileptology, Neurology, RWTH Aachen University Hospital, Aachen, Germany

^*^Corresponding author: [v.rincon.montes@fz-juelich.de](mailto:v.rincon.montes@fz-juelich.de)


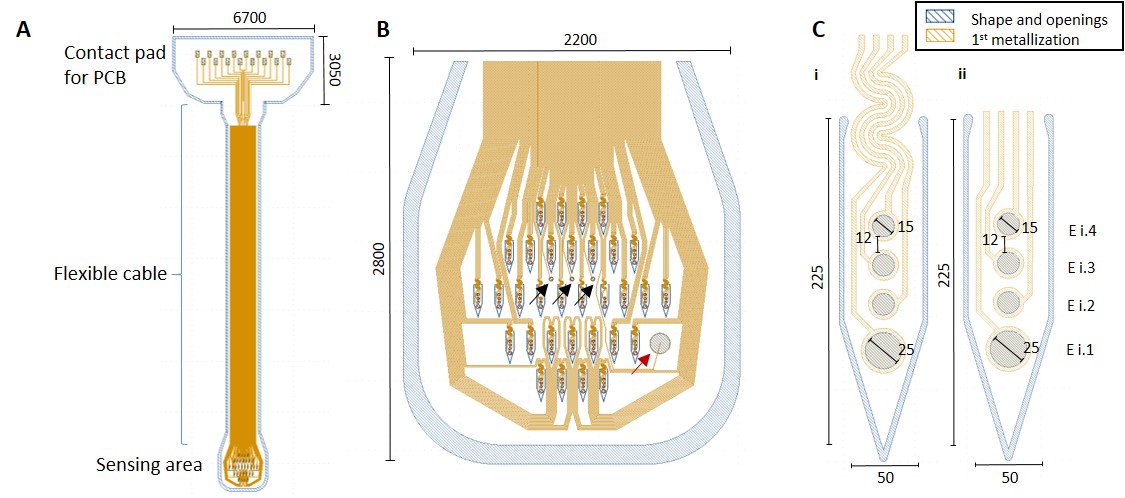


**Figure S1: 3D *kirigami* MEA design.** Exemplary design including a contact pad region, a flexible cable, and a sensing area (A) containing surface electrodes (arrows) and penetrating shanks (B), with either meander (Ci) or straight feedlines (Cii). Surface electrodes in (B) can be either used for recording (black arrows) or as an internal reference electrode (red arrow).


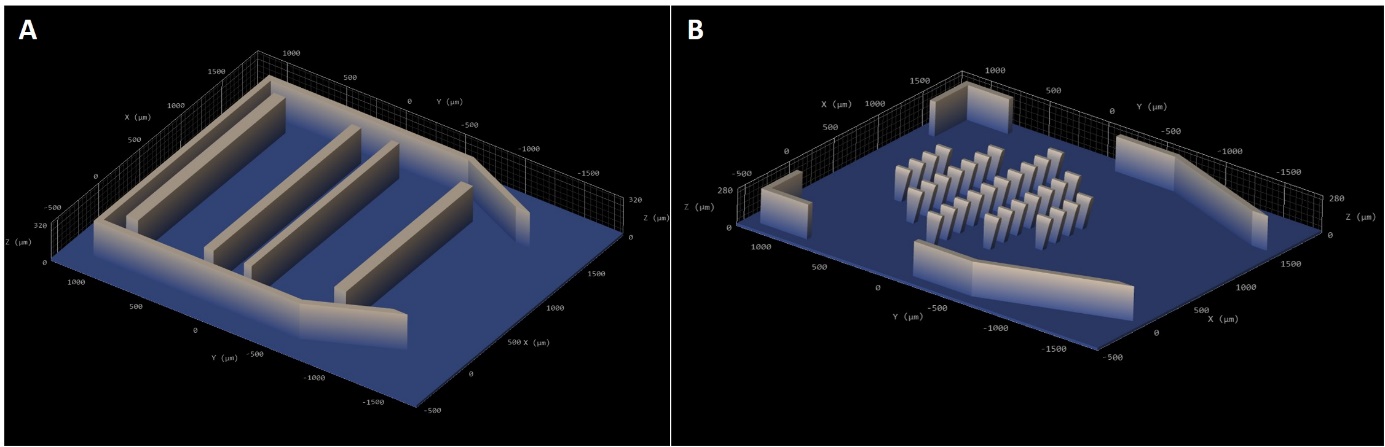


**Figure S2: Molds for *kirigami* matched die forming process.** Exemplary upper (A) and lower (B) molds for folding 2D *kirigami* MEAs with 32 shanks of a length of 225 µm. Lower and upper molds both contain sidewalls that are used as alignment structures upon folding. The upper mold (A) additionally contains elongated protruding structures to ensure homogeneous pressure during folding. The lower mold (B) contains protruding structures (blocks) which match the location of the shanks to press them out of the 2D plane.


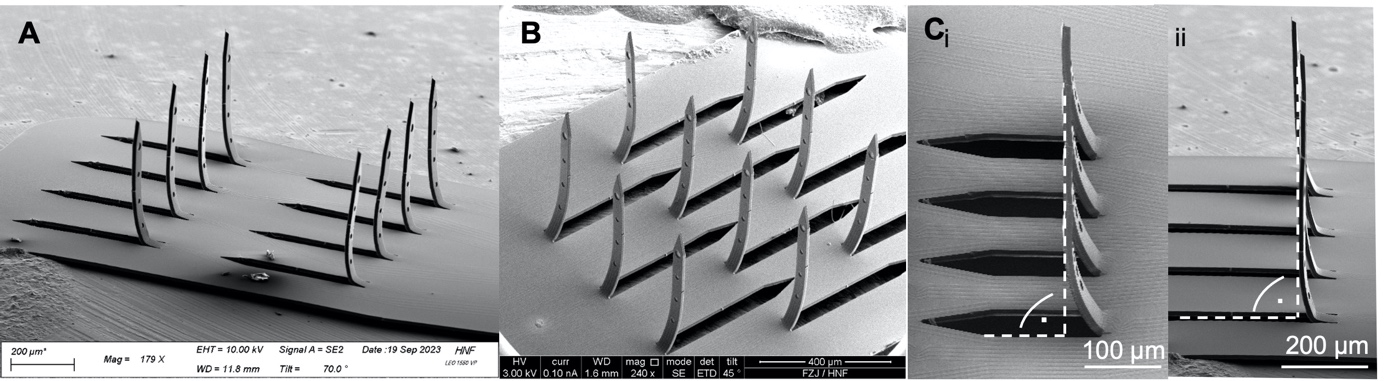


**Figure S3: 3D *kirigami* MEA designs.** Exemplary array designs with parallel shank rows (A) and diamond-shaped design (B). C) Independently of the design, the shanks are folded in an upright (〜90°C) angle (*Kiri*-225 probe in (i) and *Kiri*-500 probe in (ii)) The images were taken with a tilt of 52° and 70°, respectively. The indicated angles were measured: 89.8 ± 1.2
(N = 5 shanks) for the *Kiri*-225 samples and 86.9 ± 2.7 (N = 10 shanks) for the *Kiri*-500 samples.


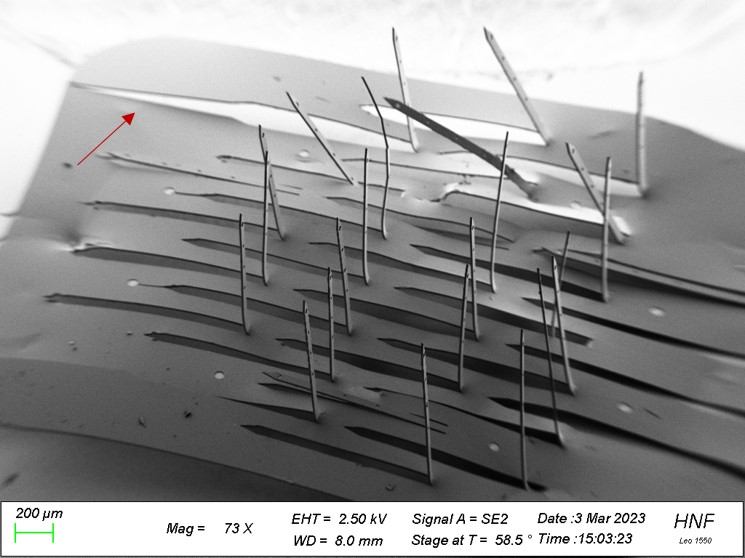


**Figure S4: Mechanical instability of 2D template in high density shank arrays.** Ripping of the 2D layout of a probe with 30 x 1000 µm long shanks and an inter-shank distance of 120 µm.

**Table S1: Bending stiffness comparison of 3D probes used in this work with 3D examples from the literature.**

| **Device** | **Components** | **Main Material** | **E-modul**  **[GPa]** | **Pillar Length [µm]** | **Diameter (at the base)**  **[µm]** | **E‧I**  **[Nµm²]** | **cross-section at centroid**  **[µm²]** | **Reference** |
| --- | --- | --- | --- | --- | --- | --- | --- | --- |
| **Silicon microneedles** | Needle electrodes | Si | 160 | 100 | 10 | 78.5 | 78.5 | Lee et al. 2022 |
| **Sea of Electrodes** | Cone-shaped needle electrodes | Si | 160 | 1200 | 20 | 398 | 177 | Zardini et al, 2021 |
| **3D printed electrodes** | Cone-shaped pillar electrodes | ormocomp | 1 | 350 | 50 | 97 | 1105 | Brown et al. 2022 |
| **3D printed and electrodeposited electrodes** | Hollow IPL multisite pillars filled with Au | IPL | 4.73 | Up to 500 | 36 x 12 | 11.59 | 250 | Abu Shihada et al., 2024 |
| **Utah Array** | Cone-shaped needle electrodes | Si | 160 | 1500 | 90 | 163040 | 3579 | Campbell et al., 1991 |
| **Michigan Array** | Flat shanks | Si | 160 |  | 10 x 50  (thickness x width) | 16667 | 500 | Wise at al., 1970 |
| **3D stacks** | Flexible stacked Michigan-style Array | Pi | 2.5 |  | 14 x 80 (thickness x width) | 45.73 | 1120 | Chung et al., 2019 |
| **In vivo Kirigami** | 4 Kirigami shanks | Pi | 2.5 | 1500 | 20 x 200 (thickness x width) | 333.3 | 4000 | Lee et al., 2022 |
| **In vitro Kirigami** | 10 Kirigami shanks | Pi | 2.5 | 1100 | 15 x 90 (thickness x width) | 63.3 | 1350 | Soscia et al., 2020 |
| **This work** | 128 Kirigami shanks | PaC | 1.7 | Up to 1000 | 10 x 50 (thickness x width) | 7.1 | 500 | *This work* |

The bending stiffness was computed by the product of the Young’s modulus *E* and the second moment of inertia *I*:

$$E\cdot I$$

Here, *I* depends on the geometry of the respective electrodes and was calculated as follows:

- For solid pillars with an outer radius *r*

$I= \frac{p}{4}\cdot r^{4}$.

- For solid cone-shaped pillars, *r* was taken at the centroid of the cone at ¼ of the height, leading to $r=\frac{3}{4}r_{b}$ with the radius *r_b_* at the base of the pillar.
- For planar shanks with a rectangular cross-section with width *b* and thickness *d*,

$$I= \frac{{db}^{3}}{12}$$

**
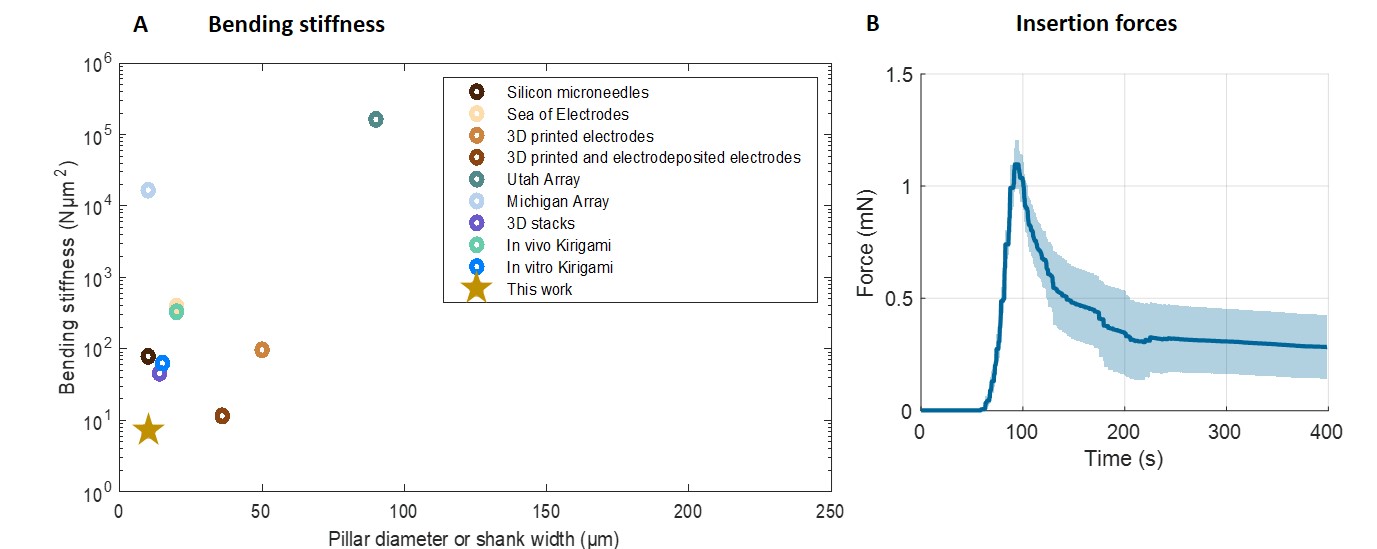
**

**Figure S5:** A) Bending Stiffness comparison of the Kirigami probes of this work with other 3D approaches. The values from the image are listed in Table S1. B) Insertion force profile of a *Kiri*-500 (N = 7) with 8 shanks penetrating agarose tissue phantom.


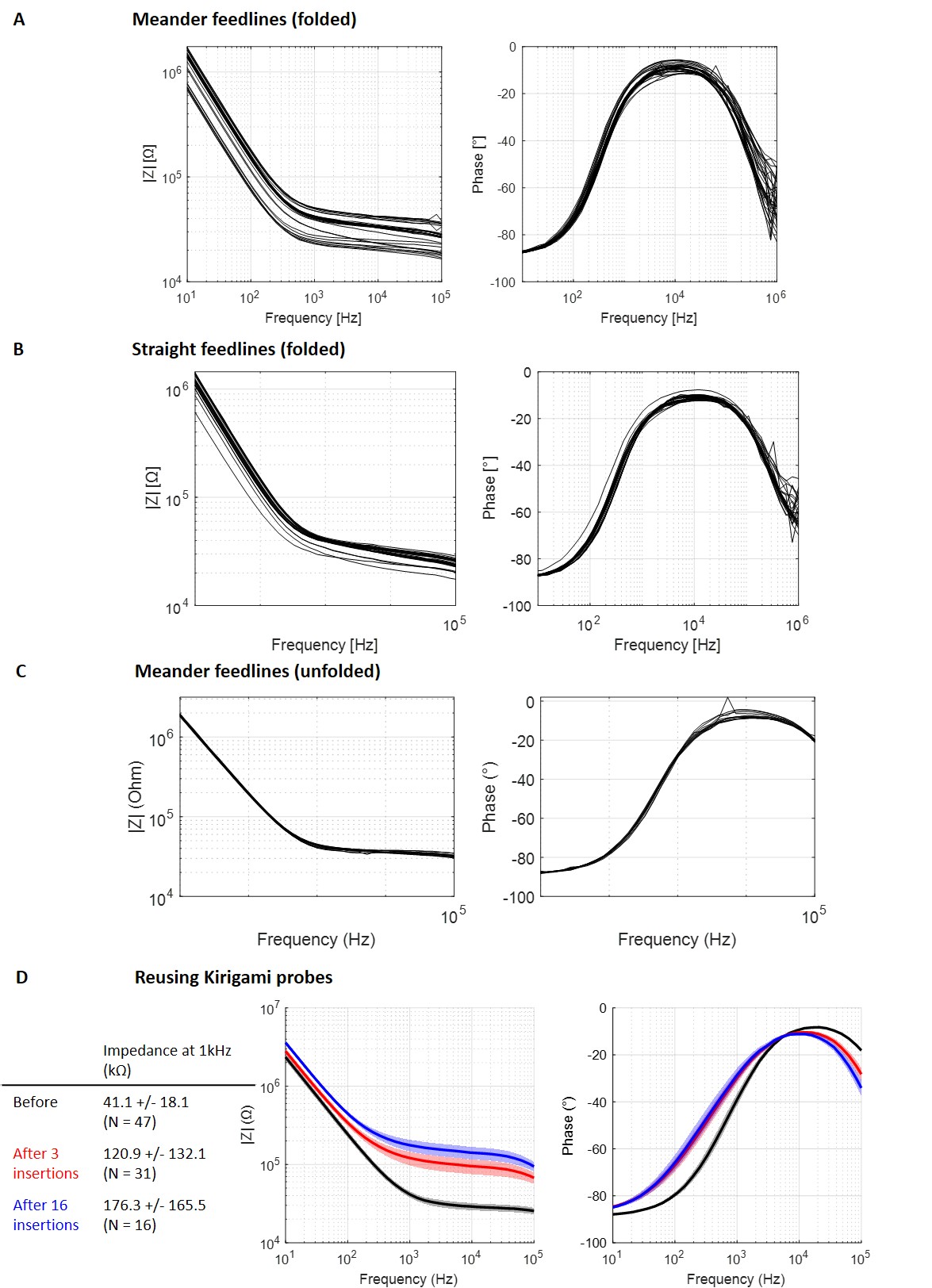


**Figure S6: Electrochemical performance of 3D *kirigami* MEAs.** Electrochemical impedance spectroscopy of *Kirigami*-based electrodes connected with meander-shaped (A) and straight (B) feedlines. C) EIS of unfolded *Kirigami* probes with meander feedlines and an electrode diameter of 15 µm). D) Impedance of reused *Kirigami*-based probes after 3 and 16 insertions in *in vitro* settings.


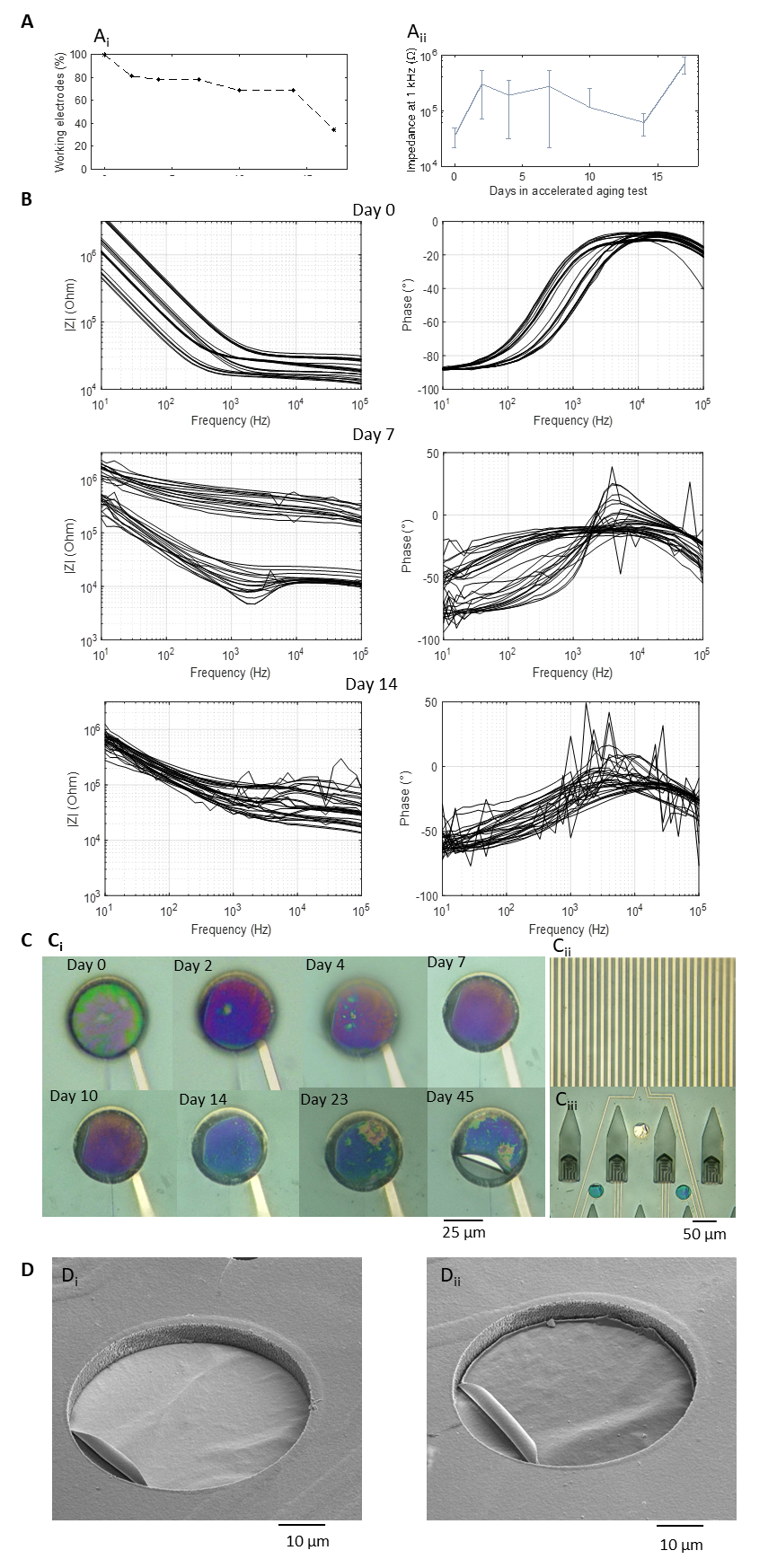


**Figure S7: Accelerated aging of 3D *kirigami* MEAs.** Accelerated aging tests were conducted with probes immersed in PBS at 60°C (*N* = 38 electrodes across three devices). A) The impedance of the electrodes was measured every two to three days and the number of working electrodes was determined using a threshold of 1 MOhm. B) Raw data of the impedance measurements at day 0, 7 and 14 of the aging test. C) Optical inspection show delamination and degradation of the PEDOT:PSS electrode coating (C_i_). There was no evidence of water entering between the PaC layers (C_ii_). Base Au electrodes were also delaminated (C_ii_). D) SEM images of the electrodes revealed that Au electrodes without (D_ii_) and with (D_i_) PEDOT:PSS delaminate. However, the Kirigami shanks are still standing in an upright position after 45 days in the accelerated aging-test.

**Table S2: Mechanical properties of untreated (no annealing), annealed at 160°C, and annealed at
200 °C 10 µm thick PaC stripes.** Values are displayed as mean +/- standard deviation. p-values < 0.05 are marked with * (N = 7 or 8 for each group, used statistical method: one-way ANOVA with 95 % confidence interval).

|  | Untreated (no annealing) | Annealed at 160°C | Annealed at 200°C |
| --- | --- | --- | --- |
| Young’s modulus  (GPa) | 1.66 +/- 0.22 | 1.70 +/- 0.32 | 2.08 +/- 0.34* (p = 0.03) |
| Stress at fracture (MPa) | 83.82 +/- 2.02 | 89.07 +/- 6.92* (p = 0.034) | 91.45 +/- 6.3* (p = 0.006) |
| Strain at fracture (%) | 14.15 +/- 4.65 | 13.93 +/- 11.25 | 9.56 +/- 9.5* (p = 0.022) |
|  |  |  |  |


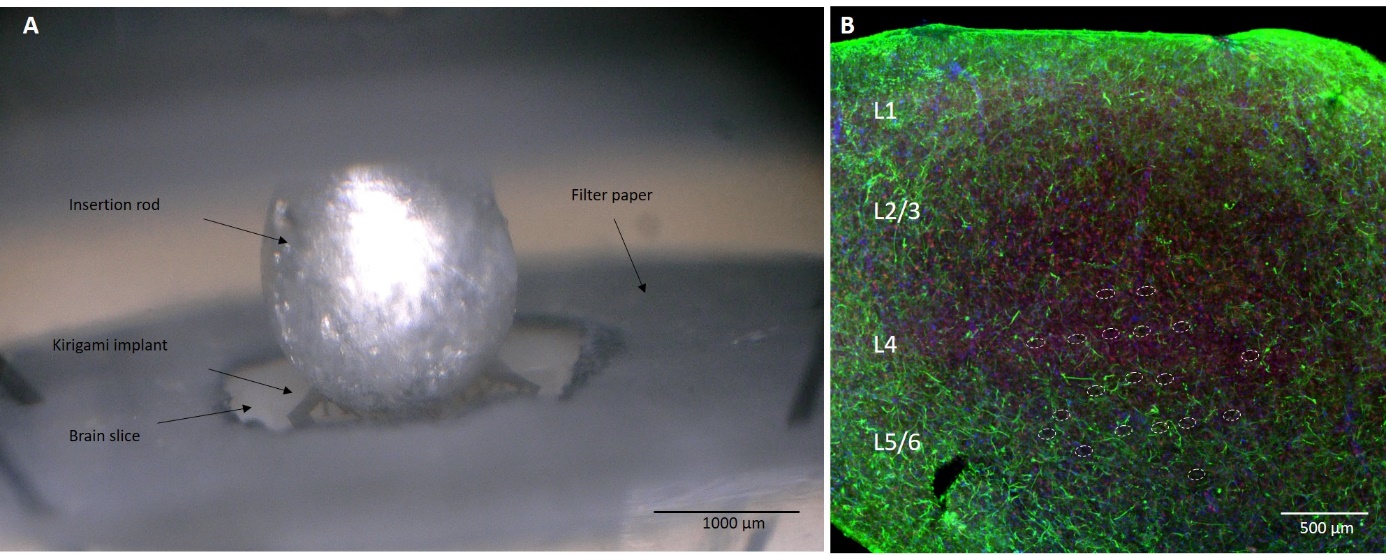
**Figure S8: Insertion of a kirigami implant into a human brain slice.** A) Insertion of a kirigami implant into an *in vitro* human brain slice using an insertion rod. The brain is fixated in a perfusion chamber with the help of filter paper and insect pins and embedded in aCSF. B) Stained brain slice and marked insertion holes (Green: GFAP, blue: DAPI, red: NeuN, 20x magnification).


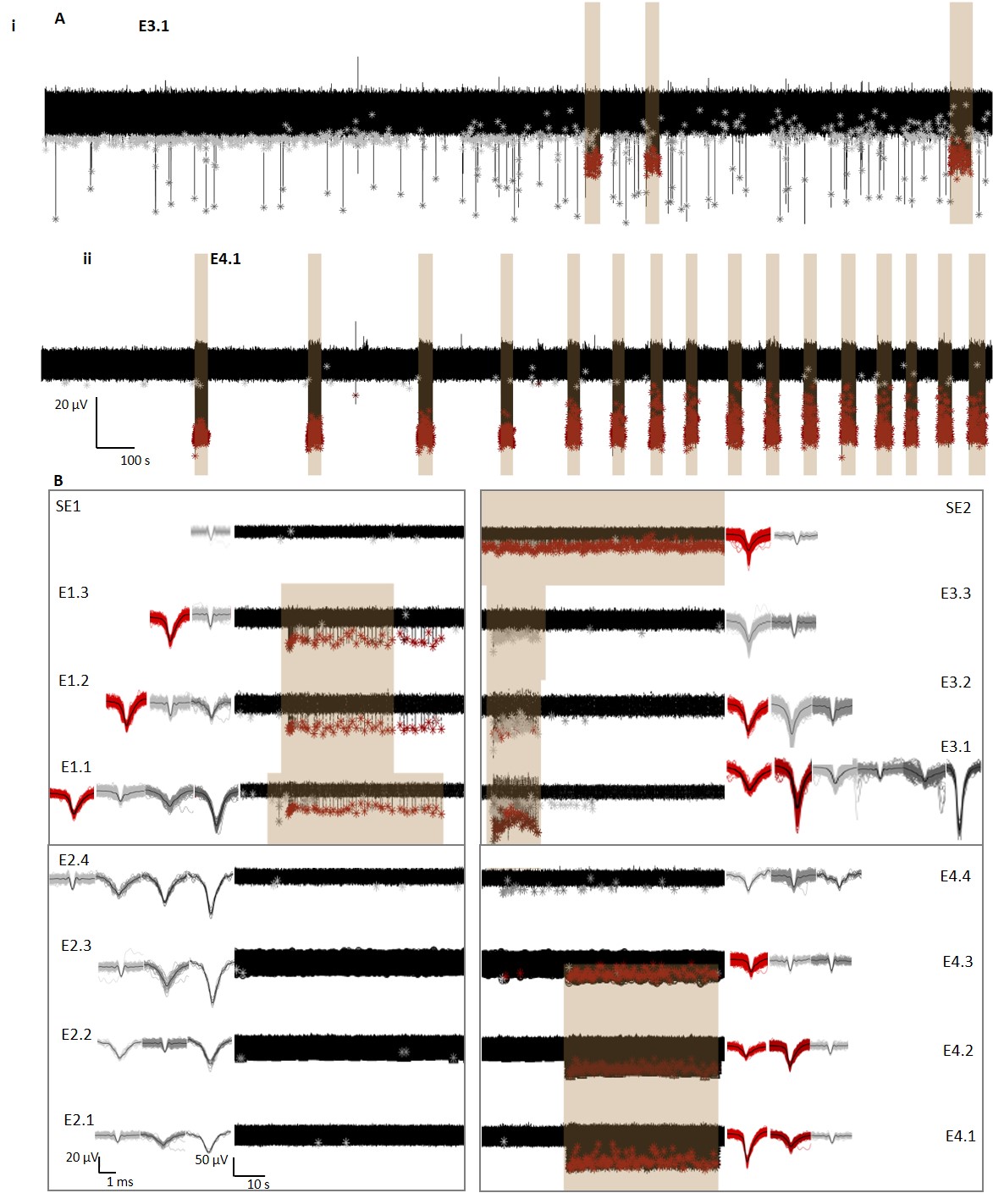


**Figure S9: Spiking activity of human brain slices in modified aCSF.** A) The complete recording of two example electrodes (E3.1 and E4.1) is displayed (bandpass-filtered data). B) Snapshot of all electrodes where SLEs are present in Shank 1, 2, 3, and 4 and in the surface electrode SE1 and SE2. The sorted spiking units are displayed next to the bandpass-filtered data. Spikes that occur during the SLEs are red, while spikes outside of the SLEs are displayed in grey. The frequency range of band-pass filtered data is 100 Hz – 3 kHz.


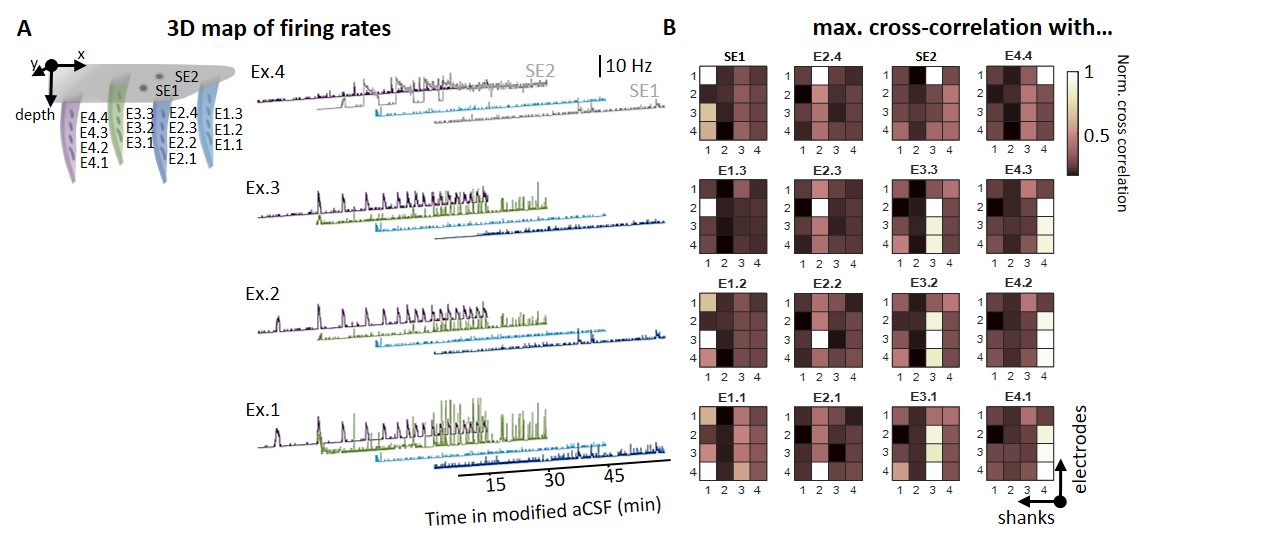


**Figure S10: Firing patterns and correlated SLEs.** Electrophysiological recordings indicate that the electrodes within one shank capture the same SLE, as indicated by an increase in spiking activity (firing rate) at specific time points, while neighboring shanks capture different events (A). This was further investigated using cross-correlations between each electrode and all other electrodes (B). Similarly, electrodes within one shank show a high cross-correlation, while electrodes of neighboring shanks are not as highly correlated.


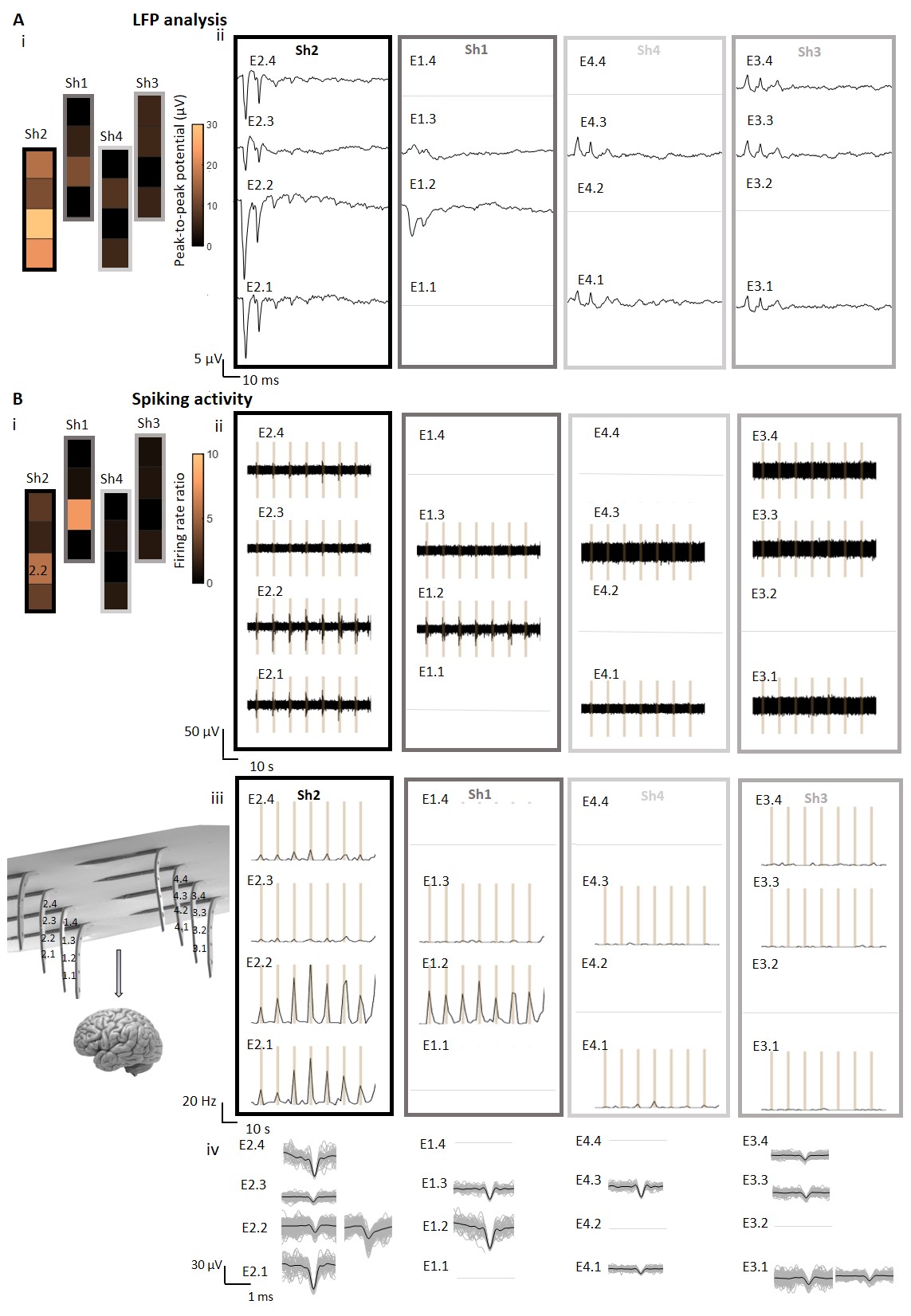


**Figure S11: 3D electrophysiological recordings of acute *in vivo* mouse somatosensory cortex.** A) Averaged LFPs show the spatial activity spread depending on the depth (z) and x-y location of the electrodes (ii). The peak-to-peak potentials were based on the averaged LFPs over 50 trials (i). B) Average changes in the spiking activity equally differs according to the electrodes position. Shown are changes in firing rates due to tactile stimulation (i), the band pass filtered data (ii), the firing rate traces (iii) and the sorted spikes (iv) for all electrodes. The firing rates rise respectively with the stimulation (brown bars). The broken electrodes are marked with grey lines.


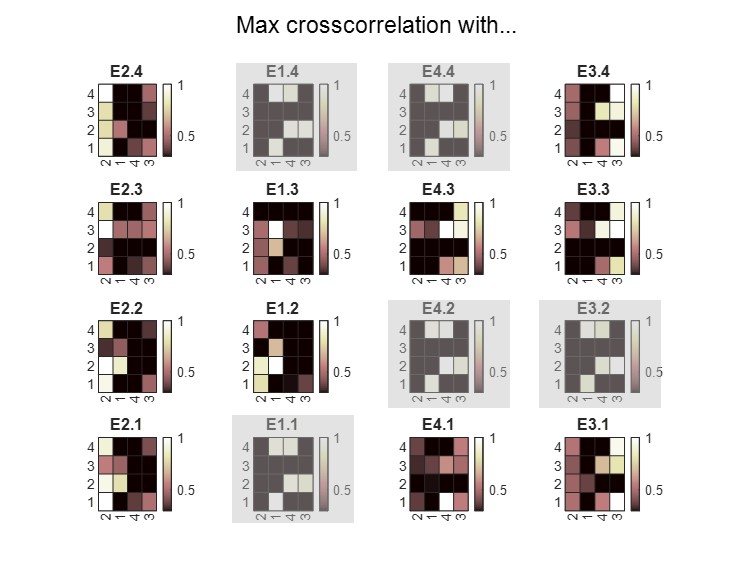


**Figure S12: Cross-correlation of the LFPs of each electrode with all the other electrodes of acute in vivo recordings in the mouse somatosensory cortex.** The maximum normalized values of the cross-correlation are displayed in each heatmap. Electrodes E3.1-E3.4 correspond to shank 3 (S3), E1.1-1.4 to shank 1 (S1), E2.1-2,4 to shank 2 (S2), and E4.1-4.4 to shank 4 (S4). The cross-correlation plots of non-working electrodes are shown in gray and as black boxes in the other heatmaps.


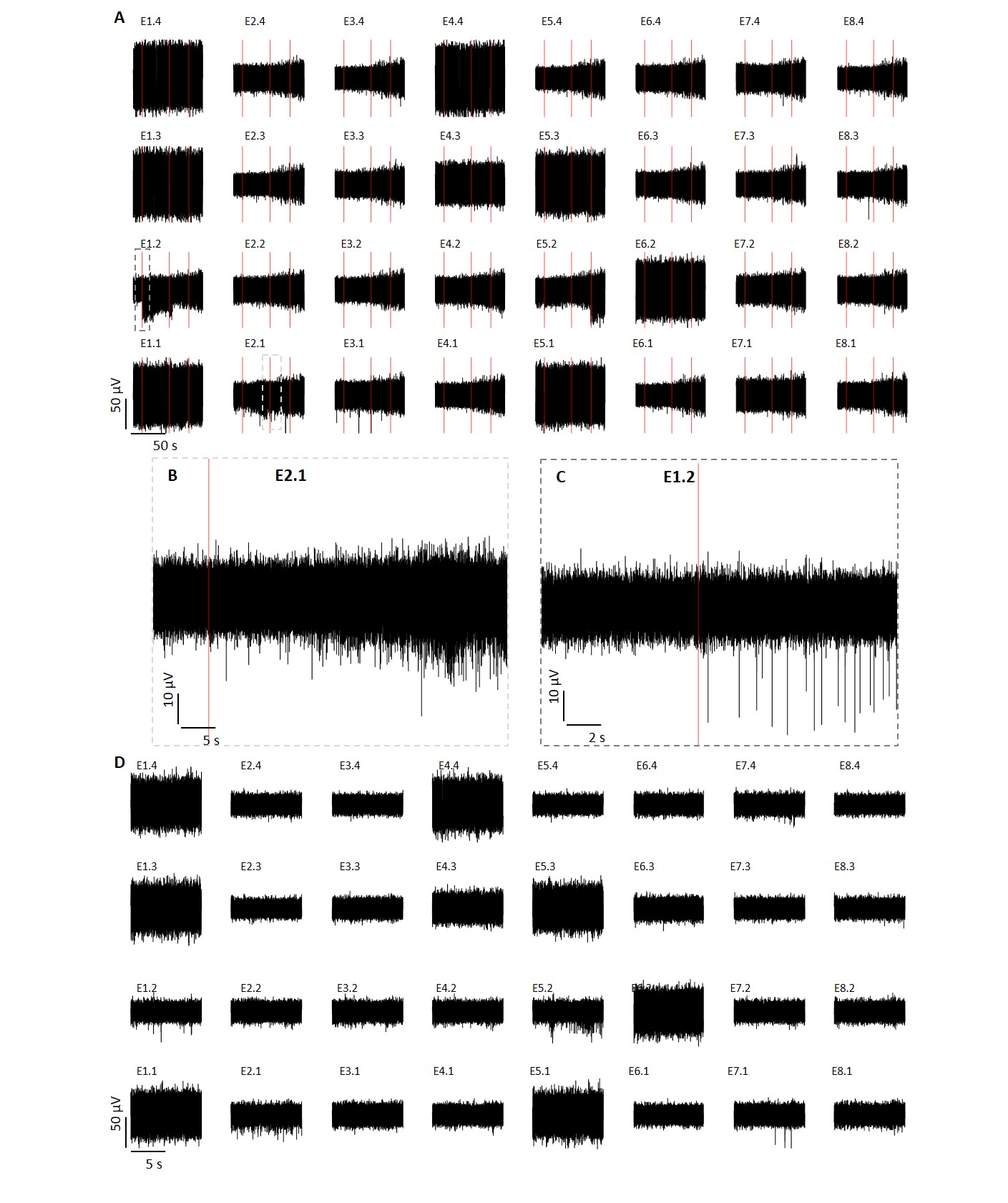


**Figure S13: Electrophysiological recordings upon implantation of a *KiRi*-500.** The implantation of a *Kiri*-500 into the mouse visual cortex. The probe contained eight shanks, each one containing four electrodes. A) Exemplary band-pass filtered (100 Hz – 3 kHz) electrophysiological data. The insertion was carried out in a stepwise manner, with the timing of each step indicated by vertical red lines. The insertion process resulted in the observation of various spiking activity patterns across shanks. While some electrodes recorded an increase in spiking activity over the subsequent seconds following insertion (as illustrated in E2.1, B), other electrodes exhibited an abrupt elevation in spiking activity (as seen in E1.2, C). Following a brief period of rest, which lasted for approximately two minutes in this example, the activity returned to its baseline state (D). This was characterized by the presence of regular spontaneous spiking activity across the majority of the electrodes (as depicted in the band-pass filtered data).


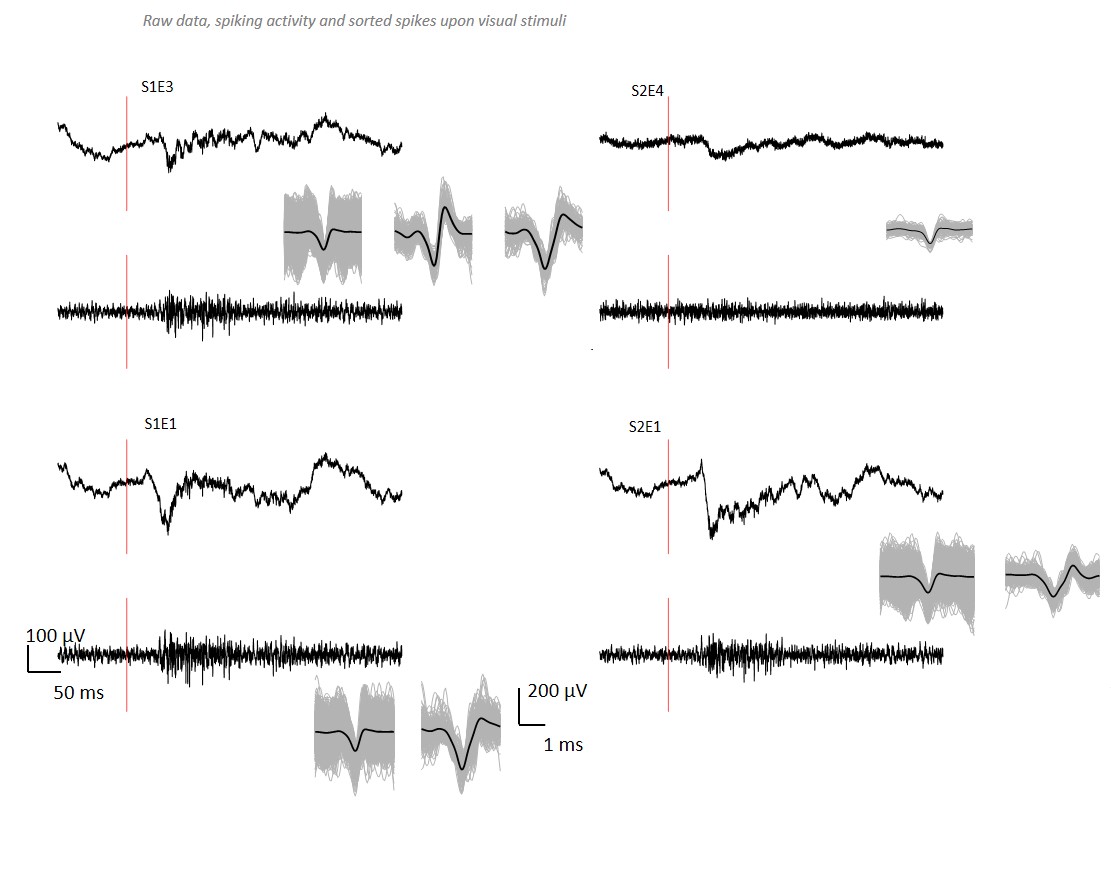


**Figure S14:** **Raw data, spiking activity (bandpass-filtered data 100 –300 kHz) and sorted spikes from recordings of the visual cortex using a *Kiri*-500 probe of selected electrodes.** The presentation of raw and filtered data exemplifies the electrophysiological activity of one trial in response to the simple grating visual stimulus, with the red bar denoting the onset of the stimulus.


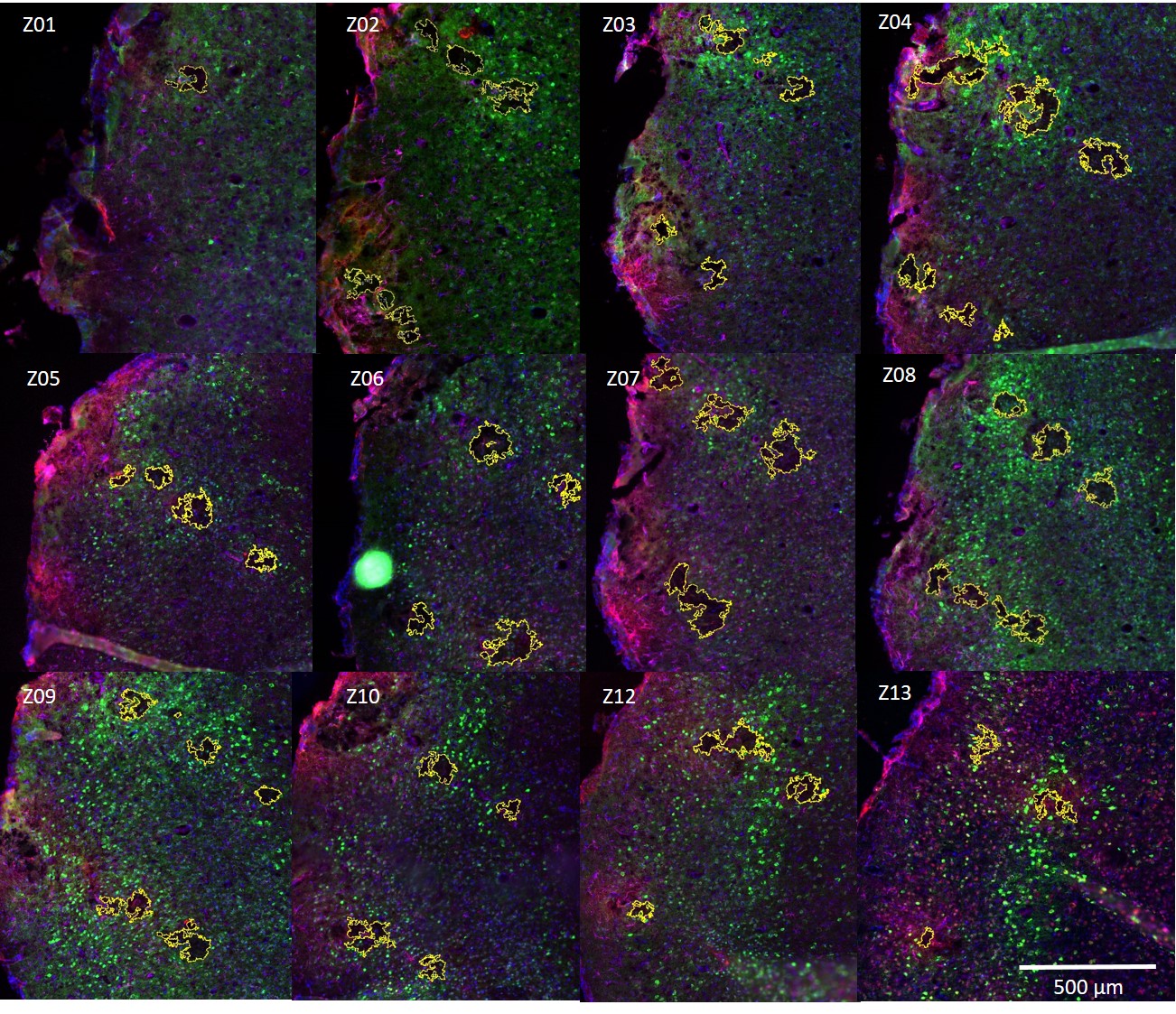
**Figure S15: Immunohistological analysis.** Stainings for mature microglia (IBA1, green), cell nuclei (DAPI, blue), microglia (GFAP, magenta) and mature neurons (NeuN, red) of 20 µm-thick horizontal slices after brain perfusion and retraction of a *Kiri*-500 implant chronically implanted for four weeks in the visual cortex of a rodent. Outlines in yellow denote the regions of interest (ROIs) enclosing the implantation lesion. Concentric or eccentric ROIs indicate a small ROI enclosing the direct implantation footprint of a shank, and a large ROI enclosing visible foreign body reactions (FBRs).

**
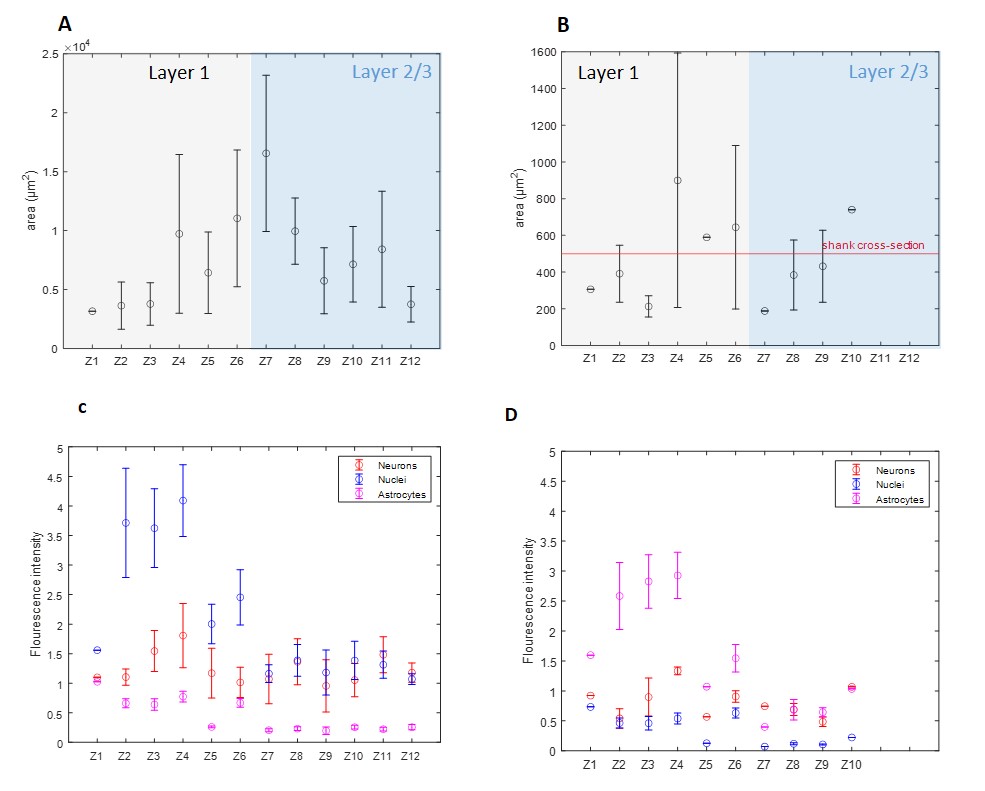
**

**Figure S16: Sizes of ROIs and relative fluorescence in immunohistological stainings.** The size of the large (A) ROIs depends on the z-depths inside the cortical layer (Z1 closest to surface). In cortical layer I, the affected regions are smaller than in layers II and III. In contrast, the size of the small (B) ROIs (insertion lesion) is smaller than the shank’s cross-sections. The relative fluorescence is overall increasing in deeper layers. Microglia in the large (C) and small (D) is getting more and more similar to the reference ROI (rel. intensity ~1), while there is also a growing amount of neurons and nuclei.


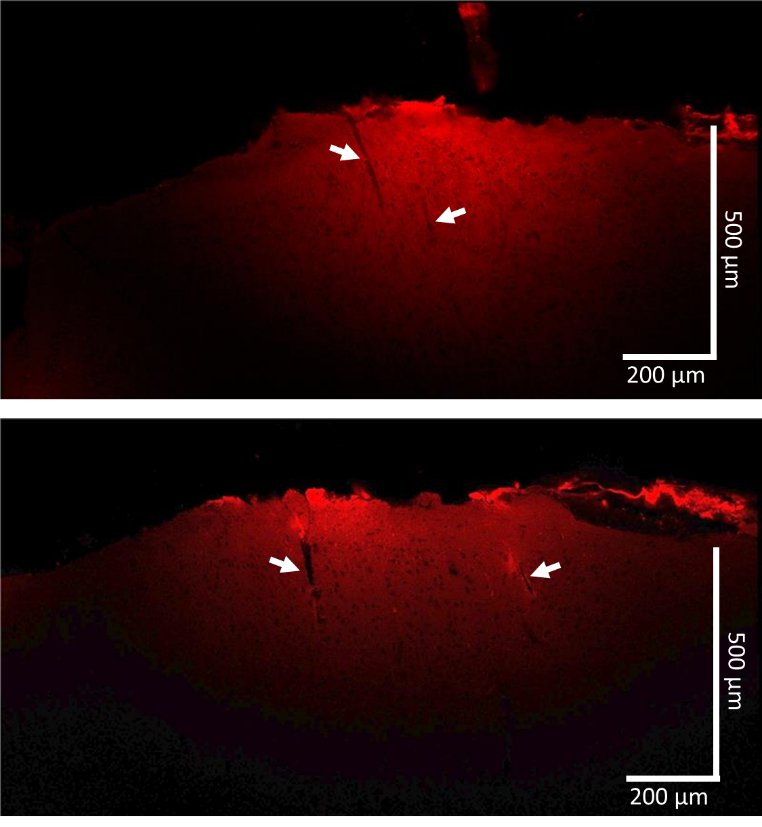


Figure S17. Insertion footprint in the cortex of a mouse. Coronal sections at the implantation site of a DiI-coated and diamond-shaped *Kiri*-500 probe acutely implanted in the somatosensory cortex of a mouse. In red DiI stained tissue. White arrows indicate insertion traces of *kirigami* shanks, demonstrating straight insertion from multiple shanks in the target region.

**Supplementary Table S3**: **Literature comparison with Utah and Michigan array as well as other *Kirigami* approaches.**

| **Publication** | **Dimension** | **Electrode count** | **Shanks** |
| --- | --- | --- | --- |
| Campbell et al, 1991 (Utah array) | \| 1500 µm length \| \| --- \| \| 90 µm diameter at the base \| | Typically 64, up to 1024 | 64 |
| Wise et al., 2004 (Michigan array) | Typically 25 – 50 µm shank width | Up to 1024 electrodes, 128 Ch | 256 |
| Takeuchi et al., 2003 | \| 1200 µm long shanks \| \| --- \| \| 160 µm wide \| \| 20 µm thick \| | 18 | 6 |
| Sim et al., 2018 | \| ~1600 mm long \| \| --- \| \| ~ 300 µm wide \| \| 7.65 µm thick \| | 64 | 16 |
| Chen et al., 2010 | \| 3500 mm long \| \| --- \| \| 200 µm wide \| \| 27.5 µm thick \| | 8 | 4 |
| Soscia et al., 2020 | \| 1100 µm long \| \| --- \| \| 90 µm wide  15 µm thick \| | Chip with up to 256 electrodes containing arrays with up to 80 electrodes | 10 |
| Lee et al., 2022 | 1500 µm length   \| ~200 µm wide \| \| --- \| \| < 20 µm thick \| | \| 24 penetrating electrodes, \| \| --- \| \| 9 surface electrodes \| | 4 |
| This work | Up to 1000 µm length  50 µm wide  10 µm thick | Up to 512 electrodes | Up to 128 |

**Supplementary videos**

**SV1** – Folding of a *KiRi*-225 probe

The video shows how a *KiRi*-225 is folded. The flexible probe is placed inside a 3D printed lower mold. When placing the upper mold on top, all shanks fold at the same time.

**SV2 –** Insertion of *Kiri*-500 probe into agarose gel.

The insertion of a *KiRi*-500 probe was tested using agarose gel which mimics the mechanical properties of neural tissue.

**SV3** – Insertion of *Kiri*-500 probe into mouse cortex.

Insertion of a *KiRi*-500 into the mouse cortex was performed after removal of the dura. The
500 µm long shanks are inserted using an insertion rod, enabling a single-shot insertion to reach layer 2/3 of the mouse cortex.
